# Disrupted Estrogen Homeostasis Drives Female-Specific Depressive-Like and Anxiety-Like Behaviors via Convergent ERα Downregulation in the Ventral Hippocampus

**DOI:** 10.7150/ijbs.137575

**Published:** 2026-09-10

**Authors:** Zhengyang Wang, Yikun Ren, Feiran Cheng, Peng Wang, Bangmin Yin, Yajie Xiang, Ying Yu, Dan Liu, Xinyi Yang, Peng Xie

**Affiliations:** 1 Department of Neurology, NHC Key Laboratory of Diagnosis and Treatment on Brain Functional Diseases, The First Affiliated Hospital of Chongqing Medical University, Chongqing, China.; 2 Jinfeng Laboratory, Chongqing, China.; 3 Chongqing Institute for Brain and Intelligence, Guangyang Bay Laboratory, Chongqing, China.

**Keywords:** depression, estradiol, ERα, sex differences, ventral hippocampus

## Abstract

Although depression exhibits prominent sex differences, mechanisms linking estrogen fluctuations to this vulnerability remain unclear. Here, we reveal that disrupted estrogen homeostasis induces depressive- and anxiety-like behaviors via convergent downregulation of estrogen receptor alpha (ERα) and subsequent neuroinflammation in the female ventral hippocampus (vHPC). In humans (n=6,481), serum estradiol (E2) showed a U-shaped association with depression, increasing risk at both extremes. In mice, while chronic restraint stress (CRS) induced depressive-like behaviors across sexes, it selectively triggered E2 deficiency, anxiety-like behaviors, and decreased vHPC ERα (but not ERβ) in females. Therefore, focusing on females, both supraphysiological E2 (sE2)-induced elevation and CRS-induced E2 deficiency produced depressive- and anxiety-like behaviors converging on vHPC ERα downregulation. Cross-species transcriptomics and multi-omics revealed global concordance between female major depressive disorder (MDD) and both mouse models, identifying ERα as a top upstream regulator associated with neuroinflammation, lipid dysregulation, and synaptic dysfunction. Pharmacological inhibition of ERα exacerbated depressive- and anxiety-like behaviors and neuroinflammation under subthreshold CRS, whereas vHPC ERα overexpression rescued CRS-induced behavioral deficits and neuroinflammatory responses. Integrating human MDD GWAS and PsyGeNET datasets supported this pathway's genetic and clinical relevance. Collectively, we identify vHPC ERα as a unifying therapeutic target for female depression.

## Introduction

Globally, depression is the primary cause of disability, affecting women almost twice as often as men, and women exhibit a differential antidepressant response compared with men 1-8. Despite decades of research, the mechanisms underlying these pronounced sex differences remain largely obscure, and no female-specific antidepressant strategies have been successfully translated into clinical practice. Thus, developing a sex-specific approach is imperative.

Notably, clinical outcomes of hormone replacement therapy (HRT) for mood regulation have been highly contradictory across real-world trials, with some reporting robust antidepressant effects while others showing increased adverse psychiatric events 9,10. In our preliminary cross-sectional study (Figure 1), serum estradiol (E2) levels exhibited a U-shaped association with depression risk, suggesting that both E2 deficiency and supraphysiological elevation may increase susceptibility to depression. This phenomenon thus underscores the critical concept of estrogen homeostasis 11. It remains unclear whether the bidirectional protective effects of estrogen conform to this U-shaped dose-dependent pattern, as well as the underlying mechanisms mediating these effects.

Understanding estrogen homeostasis requires considering the spatial and molecular complexity of the brain. While sex-based structural and functional brain differences are widely documented 12, precisely how estrogen regulates depression largely depends on its target brain regions and specific signaling pathways. Moreover, E2, the most potent endogenous estrogen, mainly delivers its neuroprotective benefits through two nuclear receptors: estrogen receptor alpha (ERα) and beta (ERβ) 13. Given their distinct brain distribution and signaling properties, a receptor- and brain region-specific approach is essential to provide deep mechanistic insights into depression 14.

Previous landmark studies have established the neuroprotective and antidepressant-like effects of physiological E2, and have partly delineated the distinct roles of ERα and ERβ 15. However, nearly all of these studies were conducted in the ovariectomized (OVX) female mouse model, which only recapitulates the acute estrogen decline following surgical oophorectomy 16. Such a model does not fully represent women with intact ovarian function and the gradual hormonal transitions of reproductive aging 17. More critically, as OVX models exclusively focus on estrogen deficiency, the specific impact of supraphysiological E2 (sE2) exposure on mood-related behaviors, as well as the receptor-specific roles of ERα and ERβ in this context, remains largely unexplored.

To address these limitations and enhance translational relevance, we employed gonadally intact male and female mice to investigate the mechanistic basis by which estrogen homeostasis regulates depressive- and anxiety-like behaviors, seeking to discover potential sex-specific treatment options for depression.

## Materials and Methods

### Association between serum E2 levels and depression risk

Previous studies have observed that lower levels of E2 could be related to a higher likelihood of depression, though few have thoroughly evaluated this connection 18,19. We focused our core analysis on male participants to achieve rigorous confounding control in endocrine epidemiology, as including female participants poses major methodological challenges from hormonal fluctuations, menstrual heterogeneity, and reproductive status 20, aligning with established high-level research paradigms 21,22. Thus, we assessed the associations between serum E2 levels and depression risk using the National Health and Nutrition Examination Survey (NHANES) 2013-2016 and 2021-2023. Based on pre-specified screening criteria, the final analysis included 6,481 participants who were eligible. Multivariable logistic regression models calculated the odds ratios (ORs) and 95% confidence intervals (CIs). Furthermore, a restricted cubic spline (RCS) model with 3 knots was conducted to examine the potential dose-response relationship. We also performed subgroup and sensitivity analyses to confirm the robustness of our findings. This research received approval from the NCHS Research Ethics Review Board, following the Declaration of Helsinki, and informed consent was obtained from all participants. Detailed methods are provided in Method S1.

### Animals

Intact male and female C57BL/6 SPF mice (7-8 weeks) were obtained from Chongqing Jilihui Biotechnology Co., Ltd. No surgical OVX or chemical castration was performed on the mice. Mice were acclimated to standard laboratory conditions (22 ± 1°C, 52 ± 2% humidity, 12-h light/dark cycle) for 1 week with free food and water. Post-acclimation, animals were randomly divided into experimental groups, stratified by matched body weight (BW) and sucrose preference (SP). Mice were subjected to chronic restraint stress (CRS) in 50 mL tubes for 6 h per day. The CRS group received stress for 21 days, and the subthreshold CRS (SCRS) group for 7 days 23, while control (CON) mice remained undisturbed. All experimenters were blinded to group assignment in the study, with only anonymized animal numbers provided to avoid subjective bias.

The Ethics Committee of Chongqing Medical University (2024-208-01) approved our research, which was carried out following the ARRIVE guidelines.

### Repeated administration of sE2 and receptor antagonist

Estradiol was purchased from MedChemExpress (Beijing, China). In this study, the dose of sE2 at 0.5 mg/kg/d was administered via intraperitoneal (I.P.) injection. This dosage was selected because it has been shown to increase peripheral E2 concentrations approximately two-fold beyond the normal physiological range in female mice 24,25, effectively simulating the hormonal state of controlled ovarian hyperstimulation. To block receptor activity, ERα antagonist MPP (purchased from MCE) was administered at 0.2 mg/kg/d via the same route. This dose was also chosen based on previous research 26.

### Ventral hippocampal AAV injections

Adeno-associated virus (AAV) vectors for ERα overexpression (AAV-ERα) were custom-produced by Vigene Biosciences (Shandong, China). Stereotaxic microinjections into the bilateral ventral hippocampus (vHPC) followed the general protocols from our previous research 27. Relative to bregma, the injection coordinates were: AP -2.8 mm, ML ± 2.6 mm, and DV -3.4/-2.6 mm, with a total volume of 500 nL per side. All subsequent experiments were initiated 3 weeks after injection.

### Behavioral tests

Behavioral tests were evaluated via the sucrose preference test (12-h SPT for anhedonia), open field test (6-min OFT for locomotion and anxiety), and tail suspension test (6-min TST for despair), following our previous protocols 28. Data were automatically analyzed via the EthoVision XT 13 software.

### Hormone assay

Mouse serum E2 levels were measured using a mouse estradiol ELISA kit (Elabscience, Wuhan, China) following the protocols.

### Estrous cycle determination and HE staining

To control for natural hormonal fluctuations, vaginal cytology was performed via hematoxylin and eosin (HE) staining on the morning of sacrifice 29. Mice were anesthetized with 20% ethyl carbamate prior to tissue collection. Female mice in the estrus stage were excluded from further analysis. However, mice in the sE2-treated group were retained, as sE2 administration inherently induces persistent estrus-like state 30. Final sample sizes are provided in Method S2.

### Real-time quantitative PCR

Total RNA extraction and real-time quantitative PCR (qPCR) were performed following our previous protocols and MIQE guidelines 27, with GAPDH as reference and 2^-ΔΔCT^ quantification. Primer sequences are listed in Table S1.

### Western blotting

Western blotting (WB) was carried out following our established methods 27. The primary antibodies used were: anti-ERα antibody (1:1000; R380695; Zen-Bioscience, Chengdu, China), anti-ERβ antibody (1:1000; 340379; Zen-Bioscience, Chengdu, China), and anti-GAPDH antibody (1:5000; 10494-1-AP; Proteintech Group, Wuhan, China). Full, uncropped images of the original western blots are provided in the separate Original Western Blots file.

### Immunofluorescence

Immunofluorescence (IF) was performed as previously described 27. Brain sections were incubated with primary antibody against ERα (1:500; ab32036; Abcam, Cambridge, UK). Images were acquired using a high-resolution slide scanner (Olympus VS200, Tokyo, Japan) in the vHPC and dorsal hippocampus (dHPC). Mean fluorescence intensity (MFI) was quantified using OlyVIA software (Olympus, Tokyo, Japan).

### Transcriptomic analysis

vHPC sampling and subsequent RNA processing were performed as previously described 31. Transcriptomic profiling was performed in two independent experimental sets: the Female CON, Female CRS, and Female sE2 groups (n = 4/group), and the CRS+ERα and CRS+GFP groups (n = 6/group). Library preparation and RNA sequencing were conducted on an Illumina NovaSeq 6000 platform by Majorbio (Shanghai, China). Detailed information is provided in Method S3.

### Proteomic analysis

vHPC sampling and subsequent protein preparation were performed as previously described 32. For the Female CON, Female CRS, and Female sE2 groups (n = 4/group), proteomic profiling was also performed by Majorbio on an Orbitrap Astral Zoom mass spectrometer. Additionally, an integrated analysis of the transcriptomic and proteomic datasets was performed to identify potential molecular mechanisms (detailed in Method S3).

### Statistical analysis

Statistical analyses were performed via R Studio 4.0.5, and *p* < 0.05 was considered significant. Values are expressed as mean ± standard error of the mean (SEM). Two-group comparisons were evaluated using Student's t-test (for normally distributed data) or the Mann-Whitney U test (for non-normally distributed data). When analyzing three or more groups, one-way ANOVA with Fisher's LSD post-hoc test was adopted for normally distributed data, and the Kruskal-Wallis H test with Bonferroni-adjusted Dunn's test for non-normally distributed data. For two-factor experimental designs, two-way ANOVA was applied, followed by LSD post-hoc tests only when significant main effects or interactions were detected. Spearman's correlation analyses were employed to test the associations between serum E2 levels, estrogen receptor expression, and behaviors.

## Results

### U-shaped association between serum E2 levels and depression

A large community-based cohort was analyzed (Figure 1A), with the sample restricted to men (n = 6,481) to minimize physiological hormonal heterogeneity 33. Detailed baseline characteristics of this study are provided in Table S2. Fully adjusted RCS plot identified a U-shaped association between log-transformed serum E2 levels and depression (*p* for nonlinear = 0.014, *p* for overall = 0.010; Figure 1B). Compared with the referent group (Q2, 18.6-24.0 pg/mL), both lower E2 levels in Q1 (OR = 1.47, 95% CI: 1.09-1.98) and higher E2 levels in Q3 (OR = 1.46, 95% CI: 1.09-1.96) and Q4 (OR = 1.36, 95% CI: 1.01-1.85) were associated with an increased risk of depression (Figure 1C). These associations were robust across subgroup and sensitivity analyses (Figures S1, S2), indicating that disrupted estrogen homeostasis, rather than merely E2 deficiency, is associated with depression risk, although further validation in independent cohorts is warranted.

### Sex-specific decreases in serum E2 and vHPC ERα mediate CRS-induced depressive- and anxiety-like behavior in mouse models

Rank-rank hypergeometric overlap (RRHO) analysis of human transcriptomic data from the ventral subiculum (a major output structure of the vHPC, hereafter referred to as vHPC; GSE102556) revealed sex-specific gene expression signatures in major depressive disorder (MDD) (Figure 2B), prompting further evaluation in intact male and female CRS mouse models (Figure 2A) 34. Female mice in estrus were excluded, and the distribution of estrous cycle stages did not differ significantly between groups (*p* = 0.4136, Figure 2C).

Twenty-one days of CRS promoted depressive-like behaviors in both sexes, as indicated by decreased SP (%) (males: *p* < 0.001; females: *p* < 0.0001) and increased immobility time in the TST (*p* < 0.05 for both sexes; Figure 2D). Notably, OFT center time was selectively decreased in females (*p* < 0.01), whereas total distance remained unaffected. Two-way ANOVA showed significant main effects of stress (*p* < 0.05) and sex (selectively for SPT and OFT, *p* < 0.05), with no significant sex × stress interactions observed (*p* > 0.05). Moreover, CRS selectively decreased serum E2 levels in females (*p* < 0.0001; Figure 2E), while no significant difference was observed in males. Spearman's correlation analyses showed that serum E2 levels were positively associated with SP (%) (*r* = 0.699, *p* < 0.01) and OFT (s) (*r* = 0.738, *p* < 0.01) in females. In males, E2 levels were positively correlated with SP (%) (*r* = 0.791, *p* < 0.001) and negatively correlated with TST (s) (*r* = -0.618, *p* < 0.05) despite no overall change (Figure 2F). Our results indicated that female mice showed higher susceptibility to the anxiety-like behavior induced by CRS. The specific decrease in the serum E2 level of females was associated with the emergence of this susceptibility, suggesting that the dysregulation of estrogen homeostasis may be the underlying cause.

To determine whether these sex-specific systemic changes translated to altered central estrogen signaling, we evaluated ER levels in the hippocampus (HPC). We identified a profound and selective downregulation of ERα, but not ERβ, at both the mRNA and protein levels in the HPC of stressed females (Figures 2G, H). In contrast, ER levels were entirely unaffected by CRS in males. Notably, this ERα deficiency was anatomically restricted to the HPC, with no significant differences detected in other stress-related brain regions (seen in Figure S3). Given the well-documented anatomical and functional specialization along the longitudinal axis of the HPC, we further assessed the spatial specificity of this ERα decrease 35,36. Strikingly, IF analyses revealed that the downregulation of ERα was anatomically restricted to the vHPC, with no significant changes in the dHPC (Figure 2I). Collectively, these results showed a sex-specific disruption of E2/ERα signaling in the vHPC of female mice.

### Disrupted estrogen homeostasis induces vHPC ERα decrease and depressive- and anxiety-like behaviors in female mice

To determine whether disrupted estrogen homeostasis directly drives depressive- and anxiety-like behaviors, we established a female sE2 mouse model alongside the CRS model (Figure 3A). Both sE2-induced supraphysiological E2 levels and CRS-induced deficiency (both* p* < 0.05; Figure 3B) produced comparable depressive- and anxiety-like behaviors. This was evidenced by decreased SP (%) in the SPT, increased immobility time in the TST, and decreased center time in the OFT (all *p* < 0.05; Figure 3C), without affecting total distance.

Strikingly, regardless of whether E2 levels were supraphysiological or deficient, a convergent decrease in vHPC ERα was observed. qPCR and WB analyses confirmed significant decreases in ERα mRNA levels (sE2: *p* < 0.05; CRS: *p* < 0.01) and protein levels (sE2: *p* < 0.01; CRS: *p* < 0.05), whereas ERβ expression remained unchanged (Figures 3D, E). Spearman's correlation analyses revealed a bidirectional relationship between serum E2 levels and vHPC ERα protein expression (Figure 3F). Under low E2 ranges (pooled CRS and CON groups), ERα levels were positively correlated with serum E2 levels (*r* = 0.905, *p* = 0.002). In contrast, under high E2 ranges (pooled sE2 and CON groups), the correlation was negative (*r* = -0.786, *p* = 0.021). Furthermore, ERα expression was consistently correlated with SP (%) (*r* = 0.706, *p* = 0.010), TST (s) (*r* = -0.888,* p* < 0.001), and OFT (s) (*r* = 0.767, *p* = 0.004) (Figure 3G). Taken together, these findings demonstrated that maintaining physiological estrogen balance is critical for vHPC ERα expression; its loss could drive depressive- and anxiety-like behaviors in female mice.

### Global transcriptomic concordance and convergent downregulation of estrogen signaling

To assess clinical relevance and identify shared molecular drivers of depression, we performed RRHO analysis. The vHPC transcriptomic profile of the female sE2 mouse model exhibited global molecular concordance with both female MDD (Figure 4A) and the female CRS mouse model (max -log10(P) = 51 and 778, respectively; Figure 4B). Gene Ontology (GO) enrichment revealed that genes concordantly downregulated across female MDD and the mouse models were predominantly enriched in synaptic vesicle processes, synapse assembly, and postsynaptic organization (Figures 4A, B).

To determine whether the shared molecular profiles are driven by disrupted estrogen signaling, we performed a targeted gene set enrichment analysis (GSEA). Notably, Estrogen-Dependent Gene Expression was significantly downregulated in the sE2 model (NES = -1.42, *p* = 0.0130; Figure 4C), mirroring the suppression of the Estrogen Responsive Gene Signature in female MDD (NES = -1.31, *p* = 0.0019; Figure 4C), and the Intracellular Estrogen Receptor Signaling in the CRS model (NES = -1.51, *p* = 0.0398; Figure 4C).

### Multi-omics integration identifies convergent biological pathways and highlights ERα as the top upstream driver

We integrated vHPC transcriptomic and proteomic analyses. Transcriptomic analysis identified 1,028 differentially expressed genes (DEGs) in the CRS group and 594 DEGs in the sE2 group (Figure 5A), while proteomic analysis revealed 355 and 319 differentially expressed proteins (DEPs) in the respective groups (Figure 5B). Global correlation analysis and clustered heatmaps further confirmed a significant consistency in expression patterns across these two molecular layers (Spearman's rho = 0.4138, *p* < 0.01; Figure S4). Integrated multi-omics analysis revealed a coordinated pathological triad comprising neuroinflammation, lipid dysregulation, and synaptic dysfunction (Figure 5C). Specifically, convergent neuroimmune activation was associated with increased inflammatory cytokine production and glucocorticoid stress responses. Simultaneously, lipid metabolism was remarkably remodeled via induced adipogenesis and suppressed structural lipid biosynthesis (e.g., glycosphingolipids and phosphatidylcholine). Finally, consistent with our prior GO analyses (Figures 4A, B), these molecular shifts were accompanied by widespread synaptic disruption, highlighted by the downregulation of postsynaptic density organization, axonogenesis, and GABAergic signaling.

Furthermore, we focused on 147 convergent DEGs and 94 convergent DEPs to screen for the key upstream regulators. Transcription factor enrichment analysis (TFEA) consistently ranked ERα (ESR1) as a top upstream regulator across both transcriptomic and proteomic datasets. Specifically, ERα ranked third and second in the transcriptomic and proteomic TFEAs, respectively (Figure 5D). Collectively, these findings identified ERα deficiency as a conserved molecular signature of female depression, nominating ERα as a key target for functional validation.

To substantiate the neuroinflammatory component of the pathological triad, we verified that both the sE2 and CRS models significantly upregulated the mRNA levels of TNF, IL-1β, and IL-6 in the vHPC of female mice (all* p* < 0.05; Figure S5A).

### ERα-antagonist MPP increases the susceptibility to depressive- and anxiety-like behaviors in female mice

To investigate the specific role of ERα in female stress vulnerability, we subjected female mice to a 7-day SCRS paradigm, a subthreshold stressor, in combination with daily administration of the selective ERα antagonist MPP or vehicle 37 (Figure 6A). qPCR and WB analyses revealed that neither SCRS nor MPP treatment significantly altered the mRNA or protein expression levels of ERα and ERβ, indicating that the antagonist exerted its effects solely by functionally blocking the receptors (Figures 6B, C). In the SPT, SCRS alone did not significantly decrease SP (%) versus the CON+Vehicle group, although a decreasing trend was observed. In contrast, MPP administration markedly reduced SP (%) compared to the SCRS+Vehicle group (*p* < 0.01; Figure 6D). In the TST, immobility time remained unaffected in the SCRS+Vehicle group versus the CON+Vehicle group, but was increased after MPP administration (*p* < 0.05). In the OFT, center time was unchanged in the SCRS+Vehicle group compared with the CON+Vehicle group, but significantly decreased in the SCRS+MPP group relative to the SCRS+Vehicle group (*p* < 0.05), whereas total distance remained unchanged. Notably, MPP alone did not induce baseline behavioral deficits (all *p* > 0.05; Figure 6D). Collectively, these results demonstrated that ERα inhibition does not independently drive depressive behaviors, but rather enhances stress susceptibility.

Moreover, MPP administration was sufficient to induce vHPC neuroinflammation following SCRS, significantly increasing TNF, IL-1β, and IL-6 mRNA levels in the vHPC of female mice (all* p* < 0.05; Figure S5B). These results highlighted ERα as a key endogenous regulator limiting susceptibility to neuroinflammatory responses.

### vHPC ERα overexpression alleviates depressive and anxiety-like behaviors and remodels the transcriptomic landscape in female mice

To investigate the specific role of ERα in the vHPC, we delivered AAV-ERα or AAV-GFP into the vHPC of female mice, followed by 21 days of CRS (Figure 7A). We first confirmed that the viral injection sites were accurately localized to the vHPC region with intact neuronal morphology (Figure 7B). AAV-ERα significantly upregulated ERα mRNA and protein expression levels, rescuing CRS-induced ERα deficits in the vHPC (Figures 7C, D). Behavioral results are shown in Figure 7E. In the SPT, the CRS+GFP group showed decreased SP (%) compared with the CON+GFP group (*p* < 0.01), whereas the CRS+ERα group exhibited increased SP (%) compared to the CRS+GFP group (*p* < 0.01). In the TST, the CRS+GFP group showed increased immobility time relative to the CON+GFP group (*p* < 0.001), whereas the CRS+ERα group exhibited lower immobility time compared with the CRS+GFP group (*p* < 0.05). In the OFT, center time was decreased in the CRS+GFP group compared with the CON+GFP group (*p* < 0.01), but was increased in the CRS+ERα group versus the CRS+GFP group (*p* < 0.05). Total distance did not differ among groups, excluding locomotor confounds. These results demonstrated that vHPC ERα overexpression could rescue stress-induced depressive- and anxiety-like behaviors in female mice.

Notably, this behavioral rescue was accompanied by the profound reversal of stress-induced neuroinflammation, as evidenced by significant suppression of TNF, IL-1β, and IL-6 mRNA levels in the CRS+ERα group relative to the CRS+GFP group (all *p* < 0.05; Figure S5C).

To further elucidate the molecular mechanisms, we also performed comparative transcriptomic analysis. ERα overexpression drove significant transcriptional changes, yielding 149 upregulated and 132 downregulated DEGs (Figures 7F, G). GSEA confirmed a profound enrichment of the estrogen receptor signaling pathway (NES = 2.01, p = 0.0015; Figure 7H), indicative of a notable functional recovery of estrogen signaling that was previously shown to be downregulated in the CRS, sE2, and human MDD datasets. Furthermore, functional enrichment analyses revealed that the ERα-regulated DEGs were significantly enriched in inflammatory, metabolic, and synaptic pathways (Figures 7I, J). To identify ERα-regulated genes involved in behavioral rescue, we intersected these DEGs with 117 convergent DEGs shared between the CRS and sE2 models. This analysis revealed 7 overlapping genes (hypergeometric test, *p* < 0.001; Figure 7K), suggesting that ERα could partially account for the transcriptomic signature associated with estrogen imbalance.

### Clinical relevance, therapeutic potential, and potential mechanism of vHPC ERα

Large-scale genetic screenings like GWAS highlighted depression's genetic vulnerability by identifying numerous pathological pathways and candidate gene targets 38. Therefore, we further performed a cross-species analysis by comparing the ERα-regulated DEGs with MDD GWAS hits (Data S1). We observed a significant overlap between the ERα-regulated DEGs and human MDD GWAS hits, yielding 10 core genes (hypergeometric test, *p* < 0.01; Figure 8A). To further examine the clinical relevance of the ERα-regulated transcriptomic profile to human MDD 39, we queried the PsyGeNET database (Data S2). Our results revealed that these ERα-regulated genes were highly enriched in mental disorders (Figure 8B). Together, these findings provided a critical theoretical foundation for investigating the targetable pathways underlying depression susceptibility.

To identify potential therapeutic strategies for female depression, we performed Connectivity Map (CMap) analysis using the ERα-regulated DEGs as the query signature 40,41. Among the mapped perturbagens, we identified several top-ranked candidate mimetics, including RU-58841 (NCS = 1.75), Physostigmine (1.73), Carbachol (1.70), Telmisartan (1.70), and Etodolac (1.70) (Figure 8C). Notably, these candidates are primarily involved in cholinergic signaling, immune-metabolic modulation, and sex hormone signaling, consistent with our prior analyses (Figure 7I). These findings provide viable pharmacological strategies for mimicking the antidepressant benefits of ERα activation and offer promising avenues for the precision treatment of female depression.

Based on these findings, we hypothesized that the estrogen homeostasis imbalance, observed in a large-scale cohort and recapitulated in female CRS and sE2 mouse models, converges on vHPC ERα downregulation as a unifying pathogenic node. This downregulation may trigger downstream neuroinflammation (e.g., elevated TNF, IL-1β, and IL-6), thereby contributing to depressive- and anxiety-like behaviors. Moreover, our cross-species analyses highlighted the genetic basis of female depression and reveal promising translational prospects (Figure 8D).

## Discussion

Our results demonstrated that the affective vulnerability to disrupted estrogen homeostasis is profoundly sex-specific. By bridging a 6,481-participant cohort with translationally relevant mouse models of stress-induced E2 deficiency and supraphysiological E2 elevation, we substantiate the estrogen homeostasis hypothesis. The observed U-shaped association reveals that both E2 depletion and excessive exposure are associated with depression. Notably, we identify a convergent receptor-specific downregulation in the vHPC, ERα but not ERβ, which serves as a shared hub linking these disparate hormonal states to depressive- and anxiety-like behaviors. These findings help explain the clinical paradox whereby distinct hormonal profiles induced similar affective behaviors, positioning vHPC ERα availability, rather than absolute serum E2 levels, as the critical gatekeeper of female emotional resilience.

For decades, mechanistic studies on estrogen and depression have relied almost exclusively on the OVX model, which suffers from two critical limitations. Firstly, the abrupt, surgically induced estrogen deficiency fails to recapitulate either the progressive trajectory of natural menopause or the stress-related E2 decline associated with depression. Secondly, existing OVX with E2 add-back (OVX+E2) paradigms rely on an artificially depleted baseline and may fail to capture the dynamic hormonal fluctuations that characterize periods of heightened affective vulnerability in females. In particular, the rapid transition from normal physiological to supraphysiological estrogen levels 42,43. To address these gaps, we established an sE2 model with an intact physiological baseline and paired it with a CRS-induced E2 deficiency mouse model to validate the estrogen homeostasis hypothesis across species 44. This approach offers profound translational relevance to adolescence and pregnancy, where depression risk paradoxically increases alongside steadily surging E2 levels 45. Collectively, our findings suggest that when E2 fluctuations exceed the estrogen homeostatic capacity of females, the ensuing downregulation of vHPC ERα may drive depressive vulnerability, supporting ERα as a pro-resilient buffer rather than a standalone driver of depressive pathology.

Once this ERα-mediated homeostatic buffer is disrupted, a cascade of downstream pathological processes ensues, primarily manifesting as sustained neuroimmune activation. Indeed, we observed significant transcriptional upregulation of pro-inflammatory markers (TNF, IL-1β, and IL-6), not only in the CRS and sE2 models but also following systemic pharmacological inhibition of ERα with the antagonist MPP. This aligns with clinical evidence showing sex-specific elevations of IL-6 in depression 46. Importantly, overexpression of vHPC ERα fully reversed these inflammatory responses. Together, these results indicate that the loss of ERα signaling induces neuroinflammation, and that restoring vHPC ERα is sufficient to mitigate this neuroinflammatory response. Furthermore, our multi-omics analyses suggest that this neuroinflammation is intertwined with lipid dysregulation and synaptic dysfunction, pointing to a potential pathological triad in which altered lipid metabolism might exacerbate synaptic impairment and depressive-like and anxiety-like behaviors.

Notably, female mice exhibited significant anxiety-like behaviors, in line with the role of the vHPC in anxiety modulation and the epidemiological finding that women are twice as vulnerable to anxiety as men 47,48. The exaggerated neuroinflammation we observed following ERα disruption provides a compelling molecular basis for these behavioral results. Indeed, neuroinflammation has been heavily implicated in the pathophysiology of anxiety, with elevated central levels of cytokines, particularly IL-6, frequently reported in individuals with anxiety disorders 49. Whether vHPC ERα serves as the critical link between estrogen homeostasis imbalance and inflammation represents a promising direction for future research into the sex differences in depression.

These findings may offer a potential explanation for the inconsistent outcomes of HRT in real-world clinical practice 50,51. We propose that the antidepressant efficacy of estrogen is contingent upon ERα availability. In cases where chronic stress or supraphysiological hormonal fluctuations have already blunted vHPC ERα expression, standard HRT may be ineffective, as the molecular 'lock' is depleted, rendering the hormonal 'key' ineffective. This not only positions ERα as a potential predictive biomarker for HRT responsiveness, but also as a prime therapeutic target. Furthermore, our cross-species analyses and CMap-identified agents, particularly those targeting cholinergic and immune-metabolic pathways, support a paradigm shift toward non-hormonal precision therapeutic strategies 52,53. Such approaches may mitigate systemic adverse effects and avoid disruption of estrogen homeostasis from exogenous hormone administration, offering a safer precision treatment strategy for females with depression.

Several limitations of the present study should be acknowledged. First, our cohort analysis was restricted to males to minimize confounding from female reproductive hormonal heterogeneity in a cross-sectional dataset 54. Consequently, future prospective longitudinal studies tracking female menstrual cycles or life-stage transitions are warranted to validate these findings. Second, although the CRS and sE2 mouse models could recapitulate core features of female MDD, these simplified surrogates simulated an extreme physiological state rather than the inherent heterogeneity. Third, although we rigorously monitored the estrous cycle of female mice to minimize hormonal variability, the potential effects of subtle cycle-dependent hormonal fluctuations on vHPC ERα signaling and behavioral results could not be completely excluded 55. Fourth, several gaps in our mechanistic understanding remain to be addressed. In particular, fully dissecting the underlying mechanisms driving the paradoxical vHPC ERα downregulation induced by sE2 (e.g., ligand-dependent receptor trafficking or epigenetic remodeling) represents a key avenue for future studies 56,57. Additionally, parallel rescue experiments were not performed in the sE2 model. Direct evidence demonstrating that overexpression of ERα reverses high-estrogen-induced affective phenotypes would position ERα downregulation as a shared causal mechanism underlying estrogen homeostasis imbalance. Fifth, the cell-type specificity of ERα in conferring vulnerability to depressive-like behaviors remains largely unexplored. Further investigations integrating single-cell transcriptomics with cell-type-specific conditional ERα knockout strategies are warranted to delineate the relevant cellular substrates 58,59. Sixth, age was not systematically examined in this study, and future work should determine whether our findings were applicable to mice with different ages 60. Seventh, while inflammatory markers were assessed only via qPCR, protein-level validation using ELISA or WB is warranted. Finally, the candidate therapeutic agents identified via CMap analysis require further validation in preclinical models and well-designed clinical trials 61.

In summary, this study demonstrates that disrupted estrogen homeostasis contributes to female vulnerability to depression through an ERα-dependent mechanism in the vHPC. These findings not only deepen our understanding of the conserved molecular mechanism underlying sex differences in depression, but also position vHPC ERα as a promising target for future precision antidepressants.

## Supplementary Material

Supplementary figures, materials and methods.

Supplementary data 1.

Supplementary data 2.

## Figures and Tables

**Figure 1:**
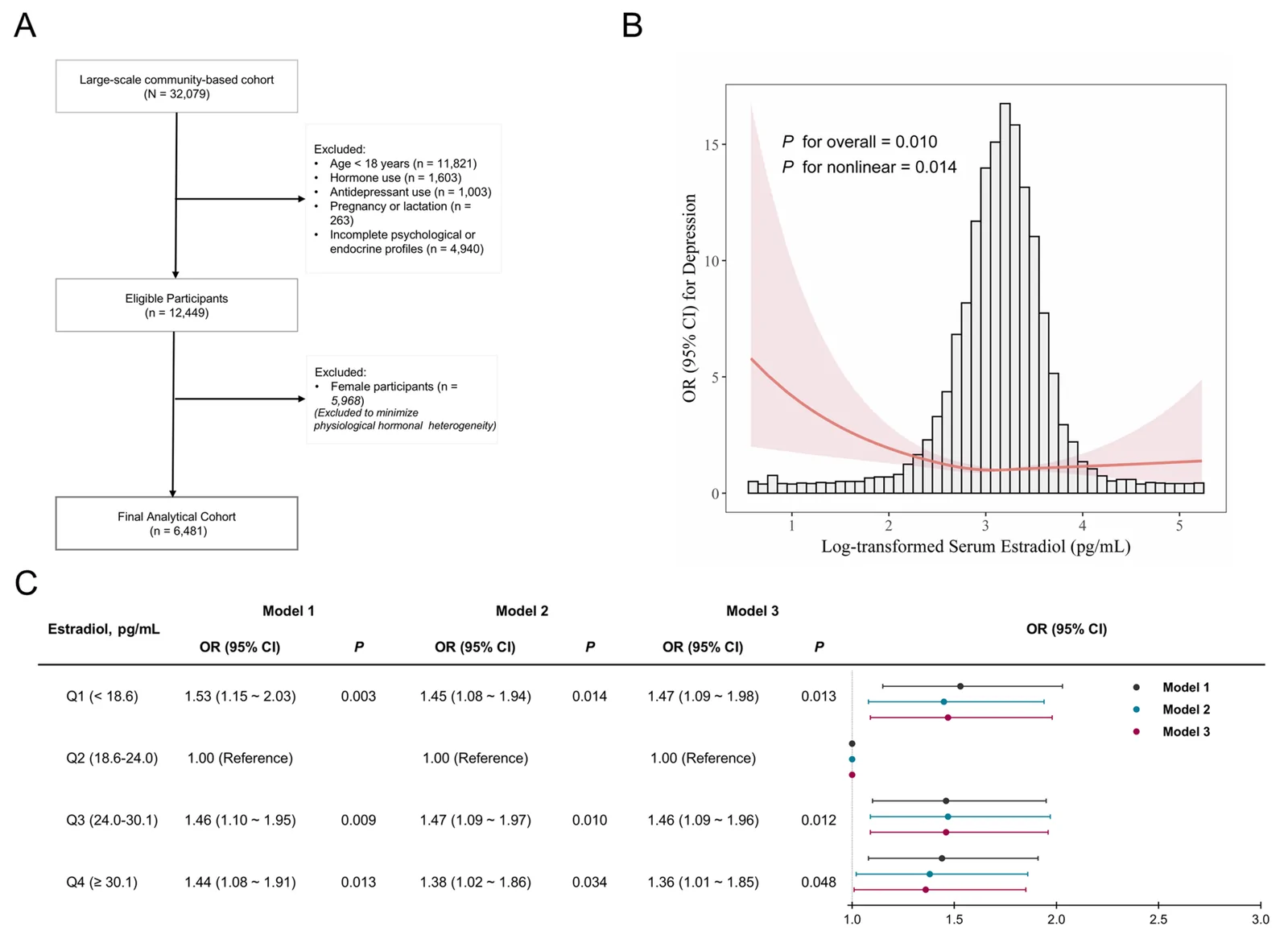
** U-shaped association between serum E2 levels and depression. (A)** Flowchart identifying 6,481 participants selected from the NHANES 2013-2016 and 2021-2023. A total of 6,481 participants were finally included. **(B)** U-shaped association between log-transformed serum E2 levels and depression using restricted cubic spline regression in multivariable-adjusted Model 3. A significant nonlinear relationship is observed, with a U-shaped curve indicating higher ORs for depression at both low and high E2 levels (*p* for nonlinear = 0.014, *p* for overall = 0.010). **(C)** Multivariable-adjusted ORs (95% CI) for depression across quartiles of serum E2 levels. Model 1: unadjusted; Model 2: adjusted for age, race, body mass index, poverty income ratio, marital status, education, smoking, drinking, physical activity, hypertension, diabetes, self-reported health; Model 3: further adjusted for log-transformed serum testosterone levels. Significant associations were observed in Q1, Q3, and Q4 compared to the reference group Q2 (all *p* < 0.05). CI, confidence interval; E2, estradiol; NHANES, National Health and Nutrition Examination Survey; OR, odds ratio.

**Figure 2:**
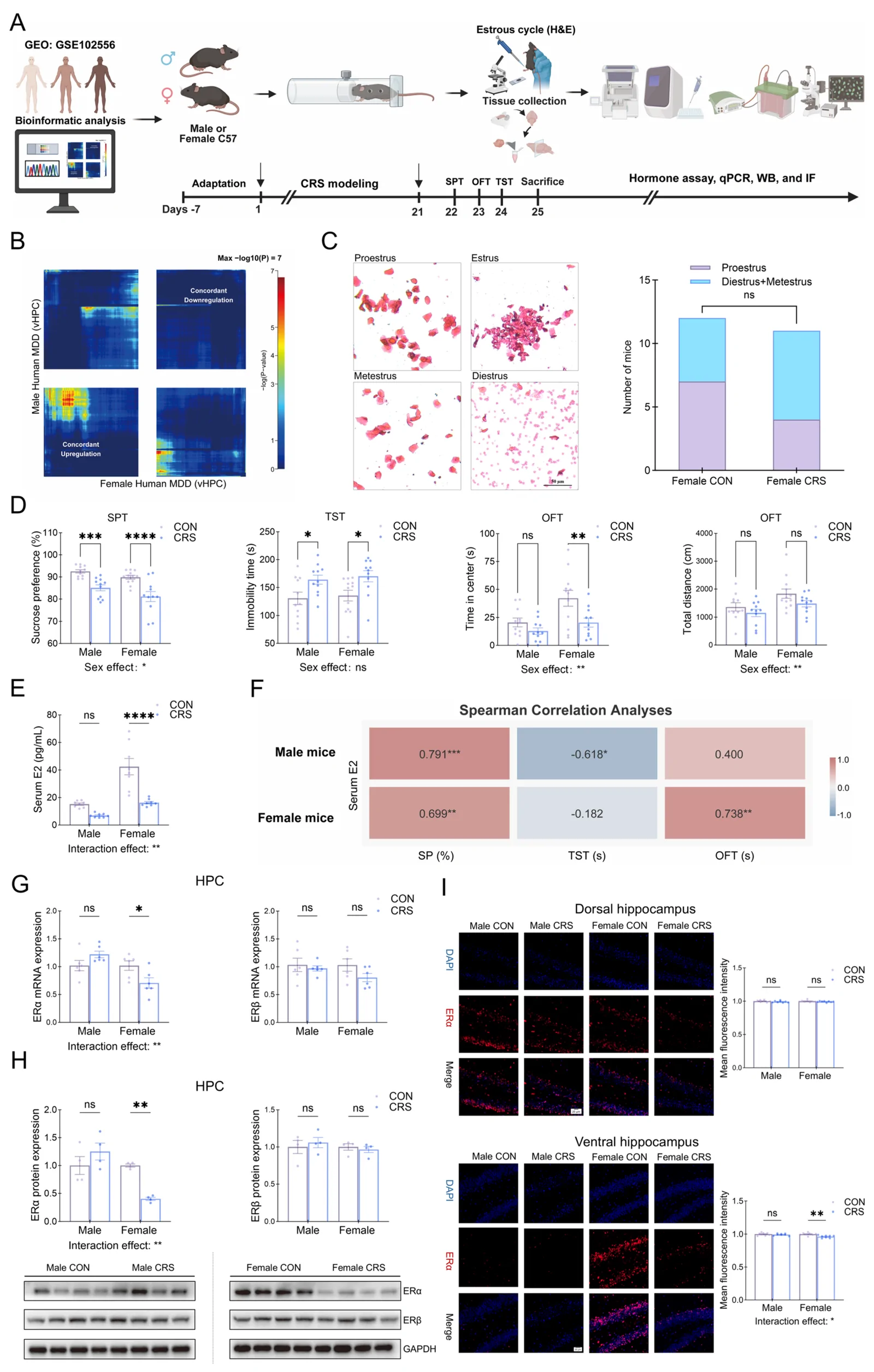
** CRS induces sex-specific depressive- and anxiety-like behaviors with decreased serum E2 and selective downregulation of vHPC ERα expression. (A)** Schematic illustration of the CRS modeling protocol in male and female mice. **(B)** RRHO analysis indicating distinct molecular signatures between sexes. **(C)** Determination and screening of estrous cycle stages in female mice. Scale bar = 50μm. **(D)** Behavioral test results after 21 days CRS modeling in male and female mice. **(E)** Serum E2 in male and female mice under CRS. **(F)** Spearman's correlation analyses between serum E2 and behavioral tests. **(G)** CRS selectively decreases HPC ERα but not ERβ mRNA expression in female mice. **(H)** CRS selectively decreases HPC ERα but not ERβ protein expression in female mice. **(I)** CRS selectively decreases vHPC ERα expression in female mice. Scale bar = 20μm. Sample sizes are n = 11-12 per group for behavior and vaginal cytology assays (panels C, D), n = 8 for ELISA (panel E), n = 6 for qPCR (panel G), n = 4 for Western blotting and immunofluorescence assays (panels H, I). Statistical significance was determined using two-way ANOVA (panels D, E, G-I), Fisher's exact test (panel C), and Spearman correlation (panel F). Values represent mean ± SEM, *p < 0.05, **p < 0.01, ***p < 0.001, ****p < 0.0001. CRS, chronic restraint stress; E2, estradiol; ERα, estrogen receptor alpha; ERβ, estrogen receptor beta; HPC, hippocampus; RRHO, rank-rank hypergeometric overlap; vHPC, ventral hippocampus.

**Figure 3:**
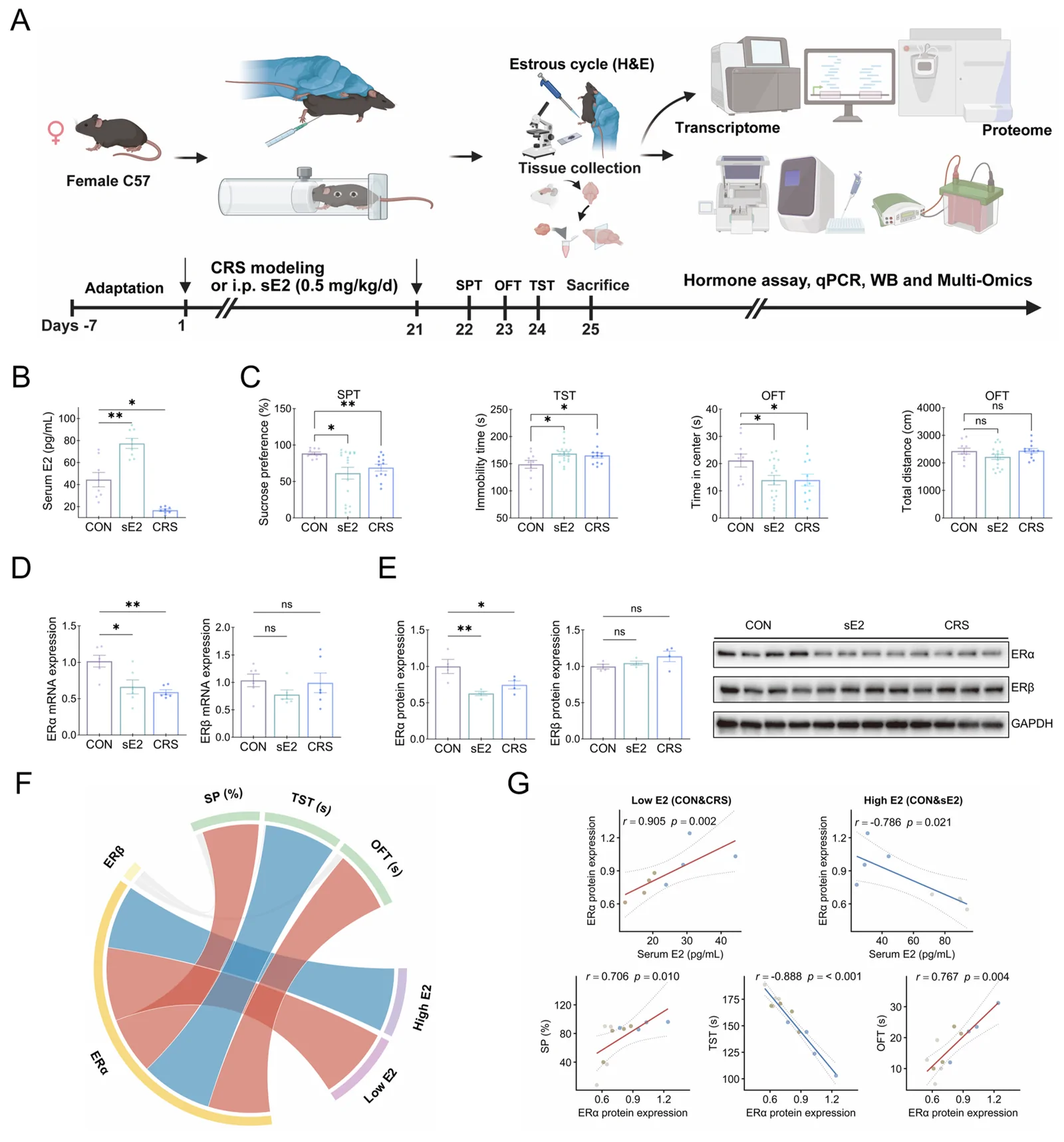
** CRS and sE2 modeling induce depressive- and anxiety-like behaviors and decrease vHPC ERα expression in female mice. (A)** Schematic illustration of the CRS modeling and sE2 administration in female mice. **(B)** Effects of sE2 administration and CRS on serum E2 levels in female mice. **(C)** sE2 administration induces CRS-like depressive- and anxiety-like behaviors in female mice. **(D)** sE2 administration and CRS selectively decrease vHPC ERα but not ERβ mRNA expression in female mice. **(E)** sE2 administration and CRS selectively decrease vHPC ERα but not ERβ protein expression in female mice. **(F-G)** Spearman's correlation analyses among serum E2, vHPC estrogen receptor expression, and depressive- and anxiety-like behaviors. Sample sizes are n = 12-20 for behavior (panel C), n = 8 for ELISA (panel B), n = 6 for qPCR (panel D), and n = 4 for Western blotting (panel E). Statistical significance was determined using one-way ANOVA (panels B-E) and Spearman correlation (panels F, G). Values represent mean ± SEM, *p < 0.05, **p < 0.01. CRS, chronic restraint stress; E2, estradiol; ERα, estrogen receptor alpha; ERβ, estrogen receptor beta; sE2, supraphysiological estradiol; vHPC, ventral hippocampus.

**Figure 4:**
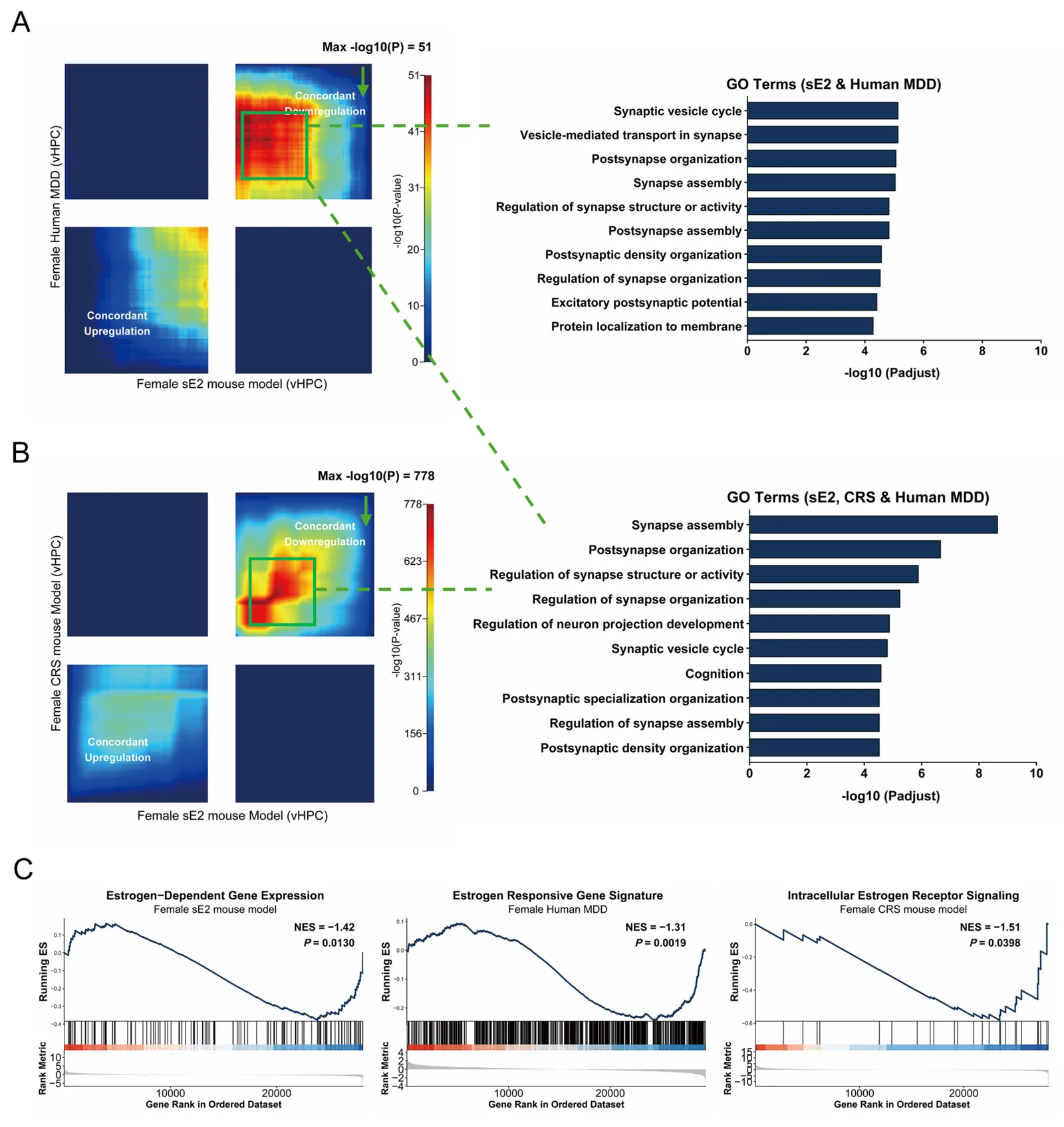
** Cross-species transcriptomic analyses reveal global concordance and convergent downregulation of estrogen signaling. (A-B)** RRHO analyses showing global transcriptomic concordance. **(A)** Comparison between the female sE2 mouse model and female human MDD. GO analysis of concordantly downregulated genes shared between female human MDD and the female sE2 model indicates enrichment in synaptic vesicle-related processes and postsynaptic organization. **(B)** Comparison between the female sE2 mouse model and the female CRS mouse model. GO analysis of genes consistently downregulated across female human MDD, sE2, and CRS models shows enrichment in synapse assembly and synaptic organization. **(C)** GSEA plots showing convergent downregulation of estrogen signaling pathways in the female sE2 mouse model, female human MDD, and female CRS mouse model. GSEA significance was assessed using permutation testing. For mouse models, n = 4 biological replicates per group. CRS, chronic restraint stress; GO, Gene Ontology; GSEA, Gene Set Enrichment Analysis; MDD, major depressive disorder; NES, normalized enrichment score; RRHO, rank-rank hypergeometric overlap; sE2, supraphysiological estradiol; vHPC, ventral hippocampus.

**Figure 5:**
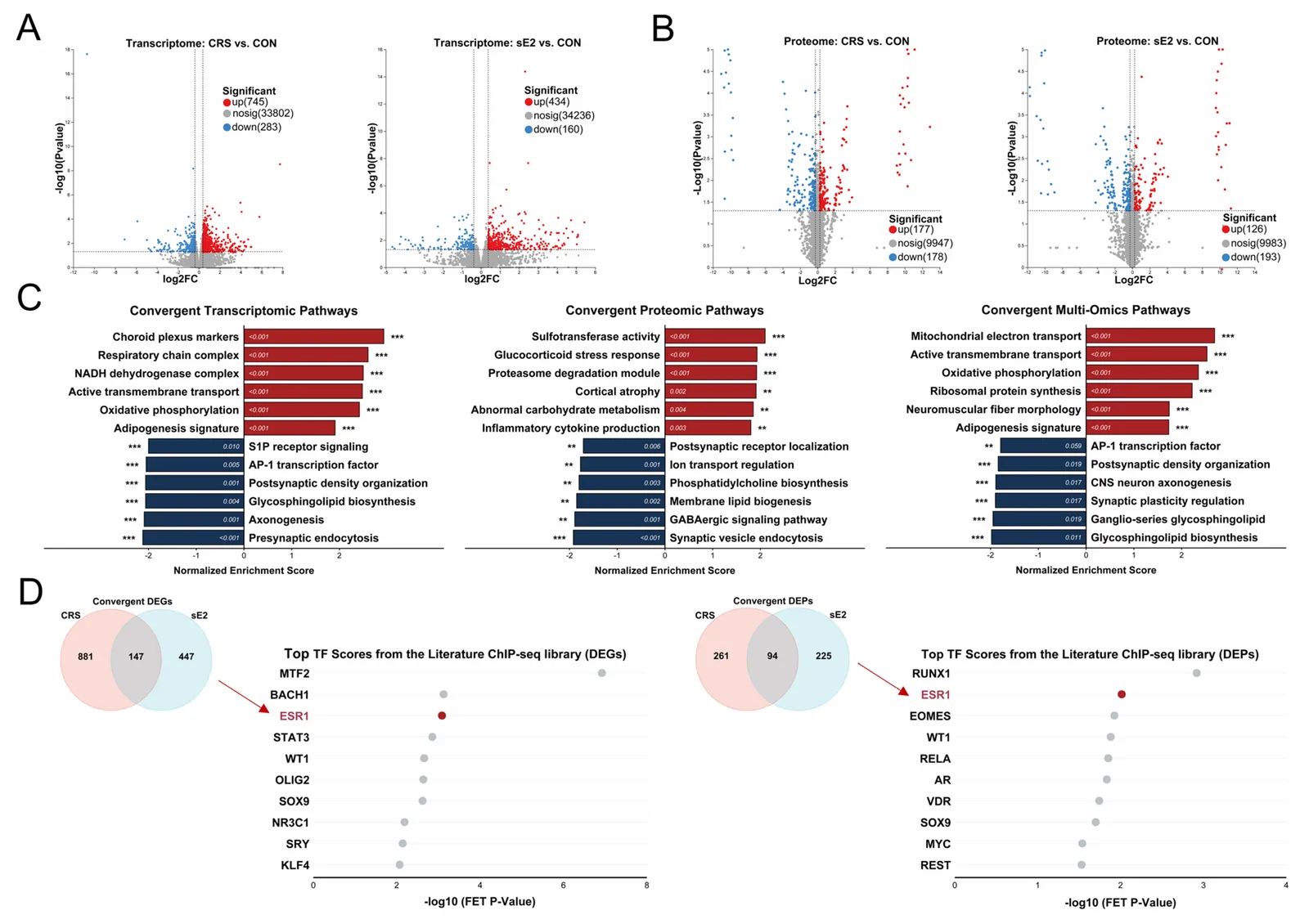
** Multi-omics integration identifies convergent biological pathways and key upstream regulators. (A-B)** Volcano plots of the transcriptome showing DEGs **(A)** and DEPs **(B)** for CRS vs. CON (left) and sE2 vs. CON (right). Significant upregulated and downregulated genes are highlighted in red and blue dots, respectively. **(C)** Bar plots displaying NES for significantly enriched pathways compared to the CON group. Red bars indicate upregulated pathways, while blue bars indicate downregulated pathways for convergent transcriptomic, proteomic, and multi-omics analyses, highlighting the neuroinflammation and the dysregulation of synaptic and lipid metabolism pathways. **(D)** TF prediction from convergent DEGs and DEPs between CRS and sE2 models. Venn diagrams show overlapping DEGs (147) and DEPs (94) used as input for TF analysis. ERα (ESR1) is highlighted as the top convergent upstream regulator. For mouse models, n = 4 biological replicates per group. Statistical significance was determined using moderated t-tests (panels A, B), GSEA permutation testing (panel C), and hypergeometric tests (panel D). CRS, chronic restraint stress; DEGs, differentially expressed genes; DEPs, differentially expressed proteins; ERα, estrogen receptor alpha; ESR1, estrogen receptor 1; NES, normalized enrichment score; sE2, supraphysiological estradiol; TF, transcription factor.

**Figure 6:**
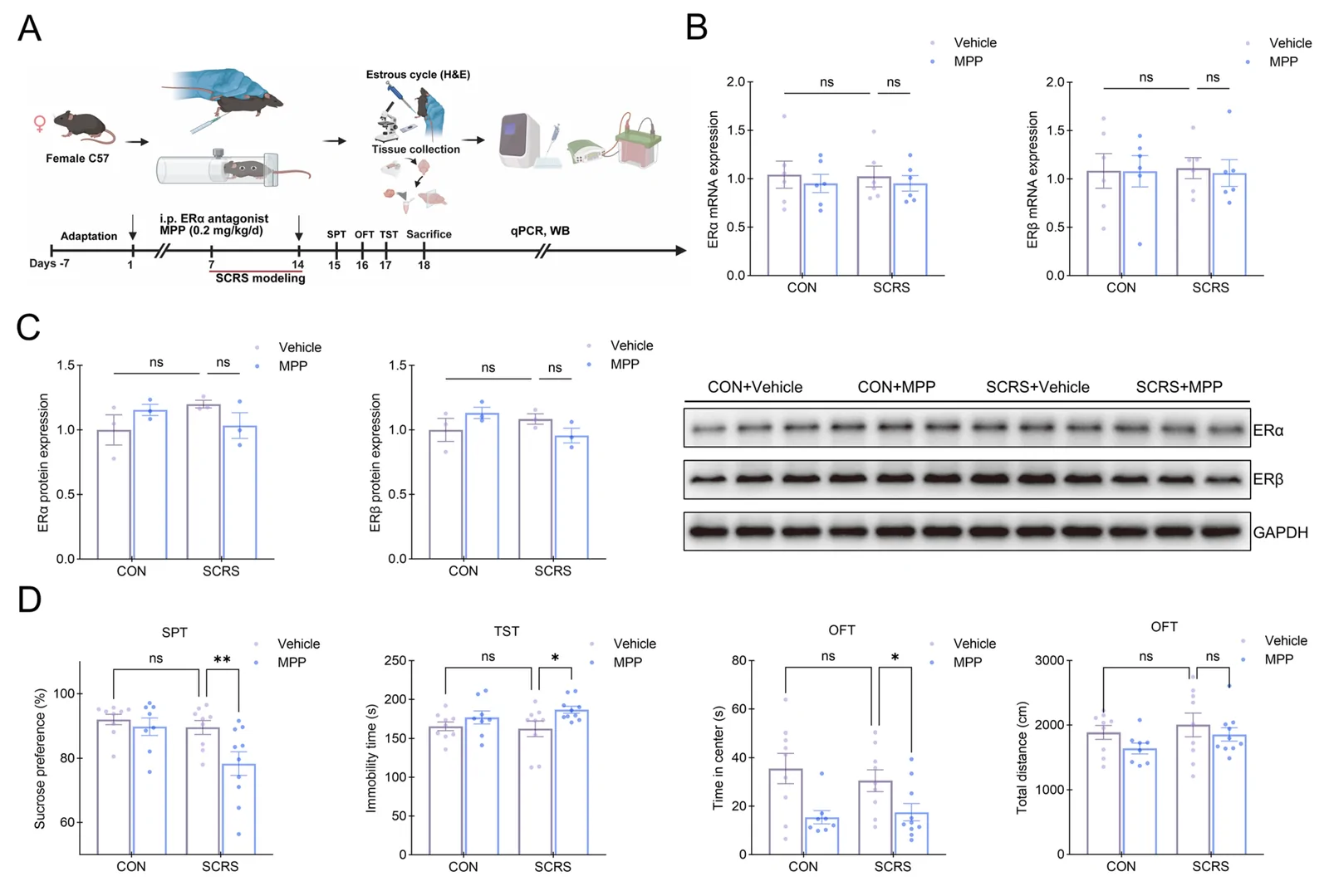
** MPP administration increases the susceptibility to depressive- and anxiety-like behaviors in female mice. (A)** Schematic illustration of the SCRS modeling and MPP administration in female mice. **(B)** MPP administration does not alter the vHPC mRNA expression of estrogen receptors in female mice. **(C)** MPP administration does not alter the vHPC protein expression of estrogen receptors in female mice. **(D)** Pharmacological inhibition of ERα increases the susceptibility to depressive- and anxiety-like behaviors in female mice. Sample sizes are n = 8-10 per group for behavior (panel D), n = 6 for qPCR (panel B), and n = 3 for Western blotting (panel C). Statistical significance was determined by two-way ANOVA (panels B-D). Values represent mean ± SEM, *p < 0.05, **p < 0.01. ERα, estrogen receptor alpha; ERβ, estrogen receptor beta; SCRS, subthreshold chronic restraint stress; vHPC, ventral hippocampus.

**Figure 7:**
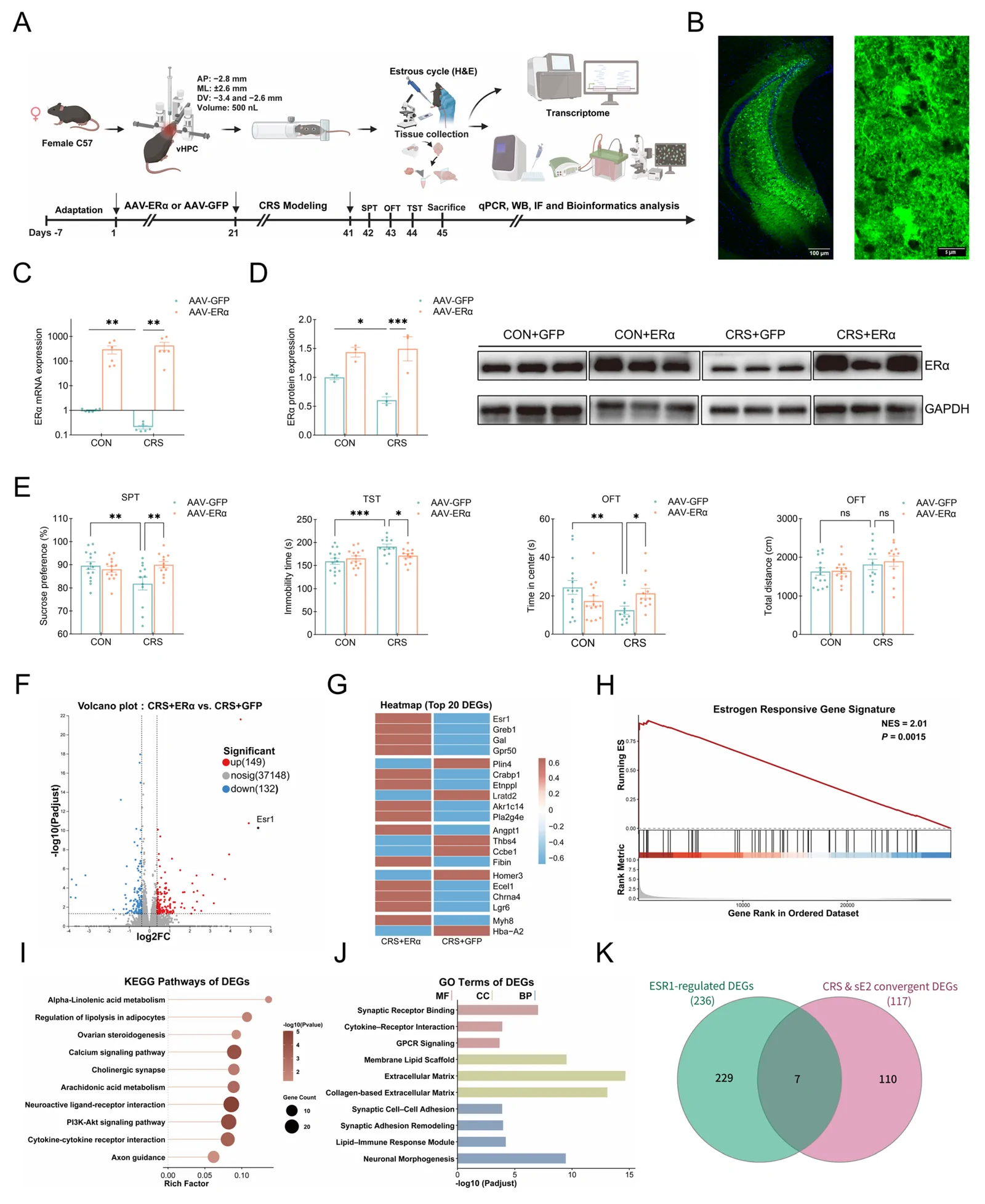
** vHPC ERα overexpression alleviates depressive- and anxiety-like behaviors and remodels the transcriptomic landscape in female mice. (A)** Schematic illustration of the experimental protocol for viral injection and CRS modeling in female mice. **(B)** Representative fluorescence images showing AAV-mediated ERα overexpression in the vHPC. Scale bars: 100μm (left) and 5μm (right). **(C)** qPCR validation of ERα overexpression efficiency (log10 scale). **(D)** Western Blot detection of ERα protein overexpression and its relative quantification. **(E)** ERα overexpression attenuated depressive- and anxiety-like behaviors in female mice. **(F)** Volcano plot of DEGs (149 upregulated, 132 downregulated). **(G)** Heatmap of top 20 DEGs associated with ERα (Esr1) overexpression. **(H)** GSEA analysis indicates a significant enrichment of the estrogen-responsive gene signature (NES = 2.01, *p* = 0.0015). **(I)** KEGG pathway enrichment analysis of identified DEGs.** (J)** GO enrichment analysis categorized by Molecular Function (MF), Cellular Component (CC), and Biological Process (BP). **(K)** Venn diagram showing the overlap between ERα-regulated DEGs and shared DEGs identified from both CRS and sE2 models (7 genes; hypergeometric test, *p* < 0.001). Sample sizes are n = 15-18 per group for behavior (panel E), n = 6 for qPCR (panel C), n = 3 for Western blotting (panel D), and n = 6 for transcriptomic analysis (panels F-K). Statistical significance was assessed using two-way ANOVA (panels C-E) and standard bioinformatics analyses (panels F-K). Values represent mean ± SEM, *p < 0.05, **p < 0.01, ***p < 0.001. AAV, adeno-associated virus; CRS, chronic restraint stress; DEGs, differentially expressed genes; ERα, estrogen receptor alpha; ESR1, estrogen receptor 1; GFP, green fluorescent protein; GO, Gene Ontology; GSEA, Gene Set Enrichment Analysis; KEGG, Kyoto Encyclopedia of Genes and Genomes; NES, normalized enrichment score; sE2, supraphysiological estradiol; vHPC, ventral hippocampus.

**Figure 8:**
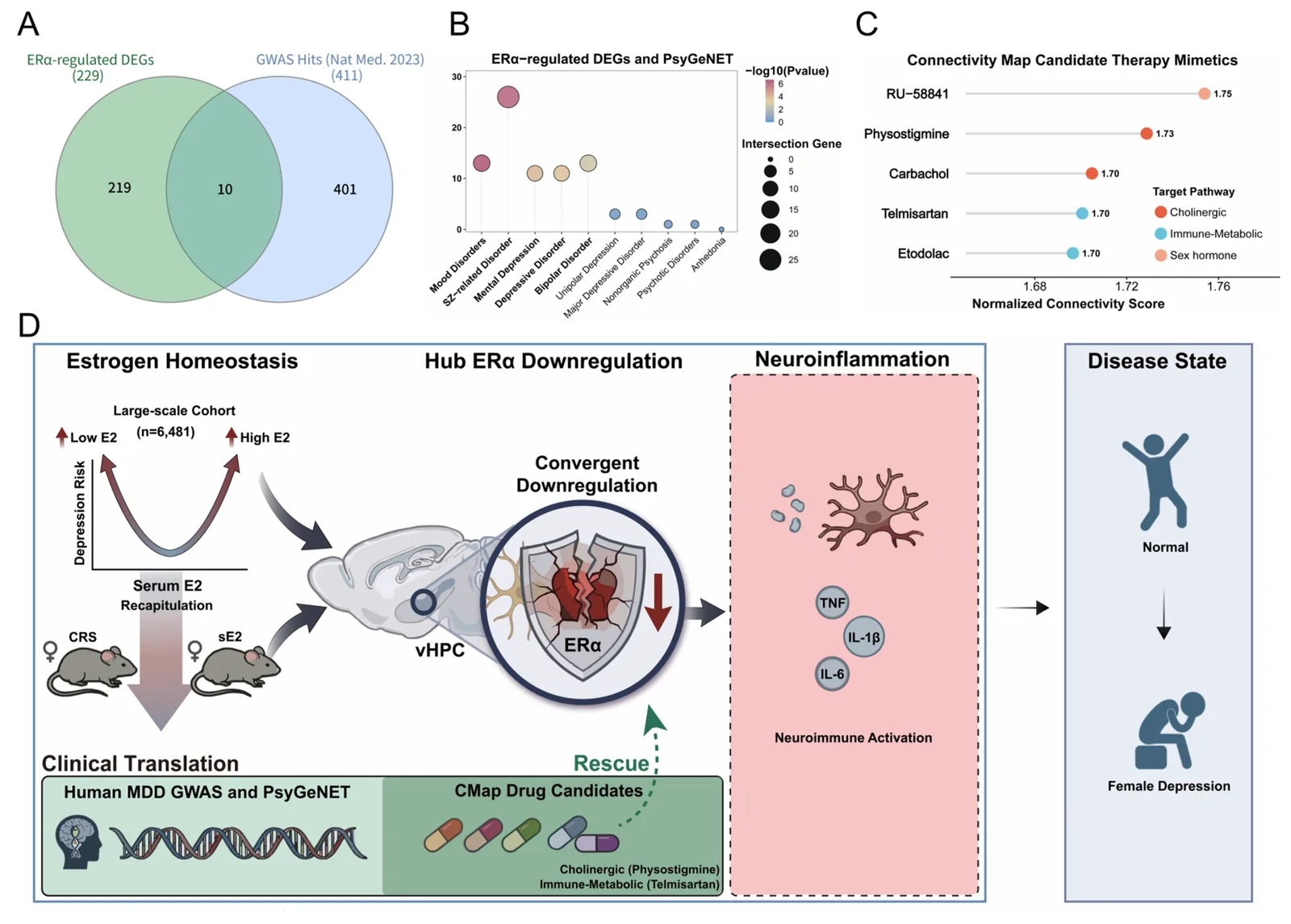
** Clinical relevance, therapeutic translation, and potential mechanism of vHPC ERα. (A)** Venn diagram showing the overlap between ERα-regulated DEGs and genes implicated in human MDD from a 2023 Nature Medicine GWAS (10 genes; hypergeometric test, *p* < 0.01). **(B)** ERα-regulated DEGs are predominantly associated with psychiatric disorders, including mood disorders and depression. Statistical significance of disease enrichment was determined using Fisher's exact test. **(C)** Connectivity Map analysis identifies top candidate drugs predicted to mimic ERα-regulated gene signatures, such as cholinergic and immune-metabolic pathways. **(D)** Summary schematic illustrating the mechanism by which disrupted estrogen homeostasis and convergent downregulation of vHPC ERα mediate female depression. DEGs, differentially expressed genes; E2, estradiol; ERα, estrogen receptor alpha; MDD, major depressive disorder; sE2, supraphysiological estradiol; vHPC, ventral hippocampus.

## Data Availability

Original data are available from the corresponding author on request. Public human (GSE102556 and NHANES) datasets are accessible via the GEO and CDC/NCHS repositories.

## Abbreviations

AAV: adeno-associated virus
CI: confidence interval
CMap: Connectivity Map
CRS: chronic restraint stress
DEGs: differentially expressed genes
DEPs: differentially expressed proteins
dHPC: dorsal hippocampus
E2: estradiol
ERα: estrogen receptor alpha
ERβ: estrogen receptor beta
GFP: green fluorescent protein
GO: gene ontology
GSEA: gene set enrichment analysis
HPC: hippocampus
IF: immunofluorescence
IL-6: interleukin-6
IL-1β: interleukin-1 beta
KEGG: Kyoto Encyclopedia of Genes and Genomes
MDD: major depressive disorder
MFI: mean fluorescence intensity
NES: normalized enrichment score
NHANES: National Health and Nutrition Examination Survey
OFT: open field test
OR: odds ratio
qPCR: real-time quantitative PCR
RCS: restricted cubic spline
RRHO: rank-rank hypergeometric overlap
sE2: supraphysiological estradiol
SCRS: subthreshold chronic restraint stress
SEM: standard error of the mean
SPT: sucrose preference test
TF: transcription factor
TNF: tumor necrosis factor
TST: tail suspension test
vHPC: ventral hippocampus
WB: western blotting
