# Supplementary material for: Disrupted Estrogen Homeostasis Drives Female-Specific Depressive-Like and Anxiety-Like Behaviors via Convergent ERα Downregulation in the Ventral Hippocampus
Source: Int J Biol Sci. 2026 Sep 10;22(14):8074–89. doi: 10.7150/ijbs.137575 (PMC13618234; doi:10.7150/ijbs.137575)
Supplement: Supplementary file 1 [file ijbsv22p8074s1.pdf]

# Disrupted Estrogen Homeostasis Drives Female-Specific Depressive-Like and Anxiety-Like Behaviors via Convergent ERα Downregulation in the Ventral Hippocampus

Zhengyang Wang[a,b,c,1], Yikun Ren[a,b,c,1], Feiran Cheng[a,b,c,1], Peng Wang[a,b,c], Bangmin Yin[a,b,c], Yajie Xiang[a,b,c], Ying Yu[a,b,c], Dan Liu[a,b,c], Xinyi Yang[a,b,c], Peng Xie[a,b,c*]

[a]Department of Neurology, NHC Key Laboratory of Diagnosis and Treatment on Brain Functional Diseases, The First Affiliated Hospital of Chongqing Medical University, Chongqing 400016, China

[b]Jinfeng Laboratory, Chongqing 401329, China

[c]Chongqing Institute for Brain and Intelligence, Guangyang Bay Laboratory, Chongqing 400064, China

*Corresponding author.

E-mail address: xiepeng@cqmu.edu.cn (P. Xie).

[1]Contributed equally

**This article contains Supplementary Tables S1–S2, Supplementary Figures S1-S5, Supplementary Data S1-S2, Supplementary Methods S1-S3, and Supplementary Original Western Blots**

**Supplementary Table S1. Primer sequences for quantitative real-time PCR**

Supplementary Table S1. Primer sequences for quantitative real-time PCR

| Gene | Forward | Reverse |
|---|---|---|
| GAPDH | CAATGACCCCTTCATTGACC | TGGACTCCACGACGTACTCA |
| TNF | GCCTATGTCTCAGCCTCTTCTC | GCCATTTGGGAACTTCTCATCC |
| IL-1β | TCTTGGGACTGATGCTGGTG | CAGAATTGCCATTGCACAACTC |
| IL-6 | GCCTTCTTGGGACTGATGCT | GCCATTGCACAACTCTTTTCTC |
| ERα | CACCAGATCCAAGGGAA | CGGCGTTGAACTCGTAG |
| ERβ | GACTGTAGAACGGTGTGGTCATCAA | CTGTGAGGTAGGAATGCGAAAC |

**Supplementary Table S2. Baseline characteristics of included participants in NHANES 2013-2016 and 2021-2023**

Supplementary Table S2. Baseline characteristics of included participants in NHANES 2013-2016 and 2021-2023

| Characteristics | Overall | Serum Estradiol, pg/mL | | | | *P* |
|---|---|---|---|---|---|---|
| | | Q1 (< 18.6) | Q2 (18.6-24.0) | Q3 (24.0-30.1) | Q4 (≥ 30.1) | |
| Participants, n | 6481 | 1606 | 1628 | 1616 | 1631 | |
| Age, years | 48.47 ± 18.47 | 49.24 ± 18.05 | 47.74 ± 18.28 | 47.94 ± 18.31 | 48.95 ± 19.20 | 0.052 |
| Body Mass Index, kg/m$^2$ | 27.80 (24.50, 31.80) | 27.15 (24.20,30.40) | 27.40 (24.40,31.10) | 28.20 (24.80,32.10) | 28.60 (24.80,33.60) | <.001 |
| Race | | | | | | 0.866 |
| Non-Hispanic White | 2923 (45.10) | 734 (45.70) | 740 (45.45) | 726 (44.93) | 723 (44.33) | |
| Other | 3558 (54.90) | 872 (54.30) | 888 (54.55) | 890 (55.07) | 908 (55.67) | |
| Smoking | | | | | | <.001 |
| Current | 1308 (20.18) | 371 (23.10) | 323 (19.84) | 313 (19.37) | 301 (18.45) | |
| Former | 1783 (27.51) | 491 (30.57) | 459 (28.19) | 418 (25.87) | 415 (25.44) | |
| Never | 3390 (52.31) | 744 (46.33) | 846 (51.97) | 885 (54.76) | 915 (56.10) | |
| Drinking | | | | | | <.001 |
| No | 1828 (28.21) | 435 (27.09) | 436 (26.78) | 433 (26.79) | 524 (32.13) | |
| Current | 4653 (71.79) | 1171 (72.91) | 1192 (73.22) | 1183 (73.21) | 1107 (67.87) | |
| Physical activity | | | | | | 0.383 |
| Inactive | 2492 (38.45) | 589 (36.67) | 629 (38.64) | 631 (39.05) | 643 (39.42) | |

| | | | | | | |
|---|---|---|---|---|---|---|
| Moderate to vigorous | 3989 (61.55) | 1017 (63.33) | 999 (61.36) | 985 (60.95) | 988 (60.58) | |
| Marital status | | | | | | <.001 |
| Alone | 2602 (40.15) | 536 (33.37) | 609 (37.41) | 671 (41.52) | 786 (48.19) | |
| Married/live with partner | 3879 (59.85) | 1070 (66.63) | 1019 (62.59) | 945 (58.48) | 845 (51.81) | |
| Education | | | | | | 0.339 |
| Above High School | 3574 (55.15) | 858 (53.42) | 897 (55.10) | 896 (55.45) | 923 (56.59) | |
| High School or Below | 2907 (44.85) | 748 (46.58) | 731 (44.90) | 720 (44.55) | 708 (43.41) | |
| Diabetes | | | | | | 0.309 |
| No | 5333 (82.29) | 1309 (81.51) | 1363 (83.72) | 1331 (82.36) | 1330 (81.55) | |
| Yes | 1148 (17.71) | 297 (18.49) | 265 (16.28) | 285 (17.64) | 301 (18.45) | |
| Hypertension | | | | | | <.001 |
| No | 3887 (59.98) | 980 (61.02) | 1030 (63.27) | 987 (61.08) | 890 (54.57) | |
| Yes | 2594 (40.02) | 626 (38.98) | 598 (36.73) | 629 (38.92) | 741 (45.43) | |
| Poverty income ratio | | | | | | 0.012 |
| < 3.0 | 3333 (51.43) | 848 (52.80) | 797 (48.96) | 807 (49.94) | 881 (54.02) | |
| ≥ 3.0 | 3148 (48.57) | 758 (47.20) | 831 (51.04) | 809 (50.06) | 750 (45.98) | |
| Self-reported health | | | | | | 0.439 |
| Very good to excellent | 2900 (44.75) | 694 (43.21) | 737 (45.27) | 725 (44.86) | 744 (45.62) | |
| Good | 2361 (36.43) | 582 (36.24) | 598 (36.73) | 602 (37.25) | 579 (35.50) | |

| | | | | | | |
|---|---|---|---|---|---|---|
| Poor to fair | 1220 (18.82) | 330 (20.55) | 293 (18.00) | 289 (17.88) | 308 (18.88) | |
| Testosterone, total (ng/dL) | 413.00 (306.00, 539.00) | 323.00 (237.19,427.00) | 393.00 (313.00,502.00) | 438.00 (334.00,563.00) | 503.00 (379.00,652.50) | <.001 |
| Depression | | | | | | 0.016 |
| No | 6026 (92.98) | 1480 (92.15) | 1542 (94.72) | 1494 (92.45) | 1510 (92.58) | |
| Yes | 455 (7.02) | 126 (7.85) | 86 (5.28) | 122 (7.55) | 121 (7.42) | |

**Supplementary Figure S1. Subgroup analyses of the association between serum estradiol levels and depression in NHANES 2013-2016 and 2021-2023**

| Subgroup | N (%) | OR (95%CI) | P | P for interaction |
|---|---|---|---|---|
| All patients | 6481 (100.00) | 0.90 (0.70 ~ 1.17) | 0.441 | |
| Age | | | | 0.115 |
| >= 65 | 1547 (23.87) | 1.38 (0.76 ~ 2.50) | 0.290 | |
| 18-65 | 4934 (76.13) | 0.82 (0.61 ~ 1.09) | 0.176 | |
| Body Mass Index | | | | 0.778 |
| >= 30 | 2273 (35.07) | 1.04 (0.68 ~ 1.60) | 0.846 | |
| < 30 | 4208 (64.93) | 0.80 (0.56 ~ 1.13) | 0.207 | |
| Race | | | | 0.785 |
| Non-Hispanic White | 2923 (45.10) | 0.92 (0.63 ~ 1.33) | 0.642 | |
| Other | 3558 (54.90) | 0.88 (0.61 ~ 1.25) | 0.465 | |
| Marital status | | | | 0.150 |
| Alone | 2602 (40.15) | 0.66 (0.46 ~ 0.93) | 0.019 | |
| Married/live with partner | 3879 (59.85) | 1.18 (0.80 ~ 1.74) | 0.413 | |
| Education | | | | 0.889 |
| Above High School | 3574 (55.15) | 0.90 (0.60 ~ 1.35) | 0.614 | |
| High School or Below | 2907 (44.85) | 0.91 (0.65 ~ 1.27) | 0.570 | |
| Poverty income ratio | | | | 0.379 |
| < 3.0 | 3333 (51.43) | 0.97 (0.71 ~ 1.33) | 0.867 | |
| >= 3.0 | 3148 (48.57) | 0.76 (0.48 ~ 1.20) | 0.238 | |
| Physical activity | | | | 0.237 |
| Inactive | 2492 (38.45) | 0.98 (0.64 ~ 1.49) | 0.925 | |
| Moderate to vigorous | 3989 (61.55) | 0.85 (0.61 ~ 1.18) | 0.325 | |
| Hypertension | | | | 0.054 |
| No | 3887 (59.98) | 0.68 (0.48 ~ 0.96) | 0.029 | |
| Yes | 2594 (40.02) | 1.29 (0.88 ~ 1.90) | 0.197 | |
| Diabetes | | | | 0.971 |
| No | 5333 (82.29) | 0.82 (0.61 ~ 1.11) | 0.202 | |
| Yes | 1148 (17.71) | 1.21 (0.71 ~ 2.03) | 0.484 | |

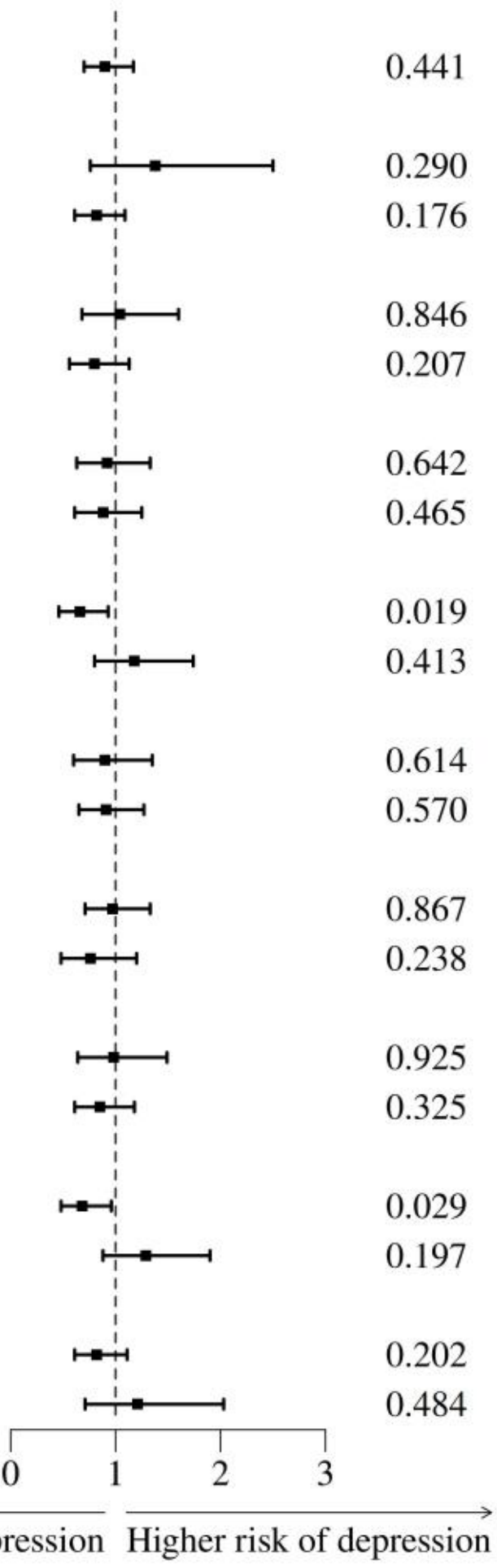


The plot shows the odds ratios (ORs) and 95% confidence intervals (CIs) per unit increment in log-transformed serum estradiol concentrations. Models were fully adjusted for age, race, body mass index, poverty income ratio, marital status, education level, smoking, drinking, physical activity, hypertension, diabetes, self-reported health, and log-transformed testosterone. Stratification variables were excluded from the corresponding models. The solid vertical line represents the reference value of 1.0. The dashed vertical line indicates the overall OR for the entire cohort.

**Supplementary Figure S2. Sensitivity analysis of the association between serum estradiol levels and depression in NHANES 2013-2016 and 2021-2023**

| Estradiol, pg/mL | Model 1 | | Model 2 | | Model 3 | |
|---|---|---|---|---|---|---|
| | OR (95% CI) | *P* | OR (95% CI) | *P* | OR (95% CI) | *P* |
| Q1 (< 18.6) | 1.53 (1.15 ~ 2.03) | 0.003 | 1.47 (1.09 ~ 1.98) | 0.011 | 1.49 (1.10 ~ 2.02) | 0.010 |
| Q2 (18.6-24.0) | 1.00 (Reference) | | 1.00 (Reference) | | 1.00 (Reference) | |
| Q3 (24.0-30.1) | 1.46 (1.10 ~ 1.95) | 0.009 | 1.53 (1.13 ~ 2.05) | 0.006 | 1.52 (1.12 ~ 2.04) | 0.006 |
| Q4 (≥ 30.1) | 1.44 (1.08 ~ 1.91) | 0.013 | 1.40 (1.03 ~ 1.89) | 0.030 | 1.38 (1.01 ~ 1.88) | 0.043 |

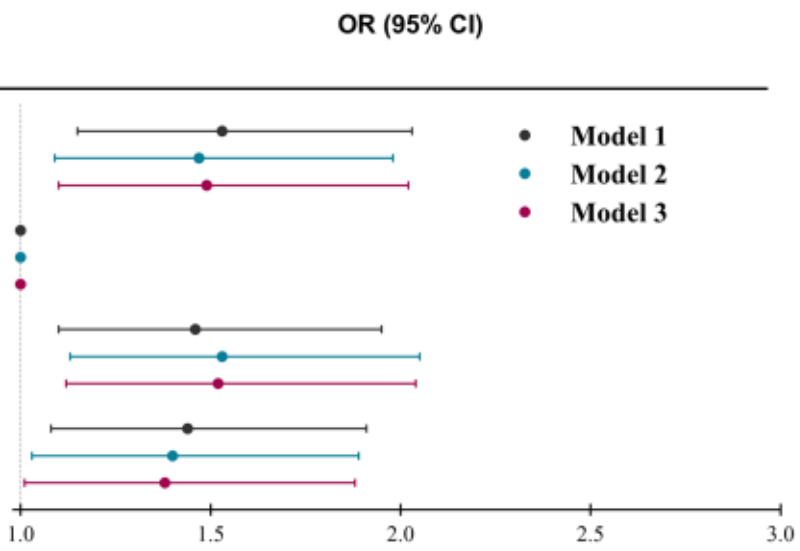


Analyses were performed using the original dataset without missing value imputation. Multivariable-adjusted ORs (95% CI) for depression across quartiles of estradiol concentrations. Model 1: unadjusted; Model 2: adjusted for age, race, body mass index, poverty income ratio, marital status, education level, smoking, drinking, physical activity, hypertension, diabetes, self-reported health; Model 3: additionally adjusted for log-transformed testosterone. Significant associations were observed in Q1, Q3, and Q4 compared to the reference group Q2 (all $p < 0.05$). OR, odds ratio; CI, confidence interval.

**Supplementary Figure S3. CRS selectively decreases ERα but not ERβ mRNA expression in the hippocampus of female mice**

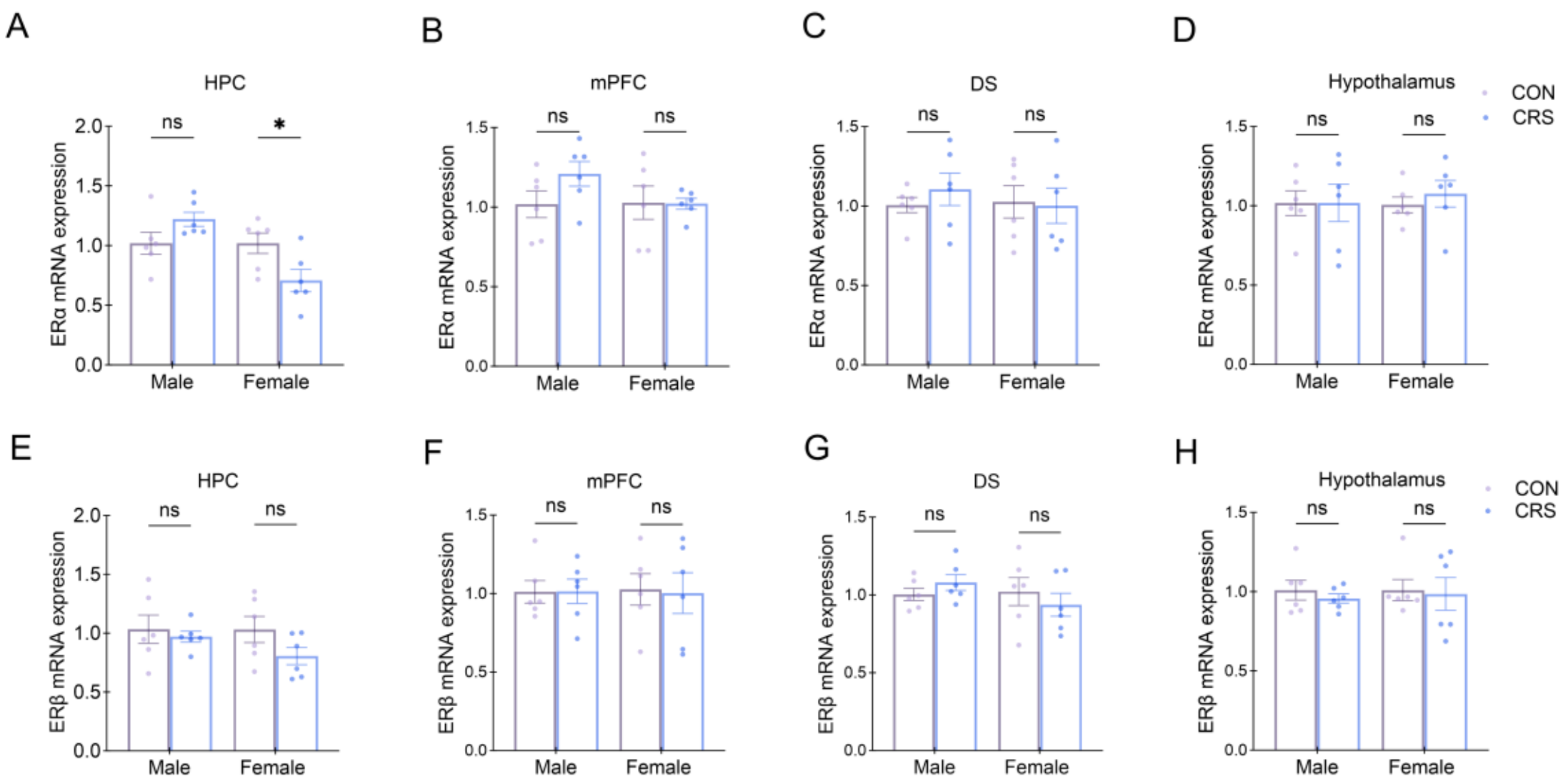


**(A–D)** ERα mRNA expression levels in the hippocampus (HPC), medial prefrontal cortex (mPFC), dorsal striatum (DS), and hypothalamus. Note the selective downregulation in the HPC. **(E–H)** ERβ mRNA expression levels in the respective brain regions. Data are presented as mean ± standard error of mean, *$p < 0.05$.

**Supplementary Figure S4. Integrated analysis of the transcriptome and proteome**

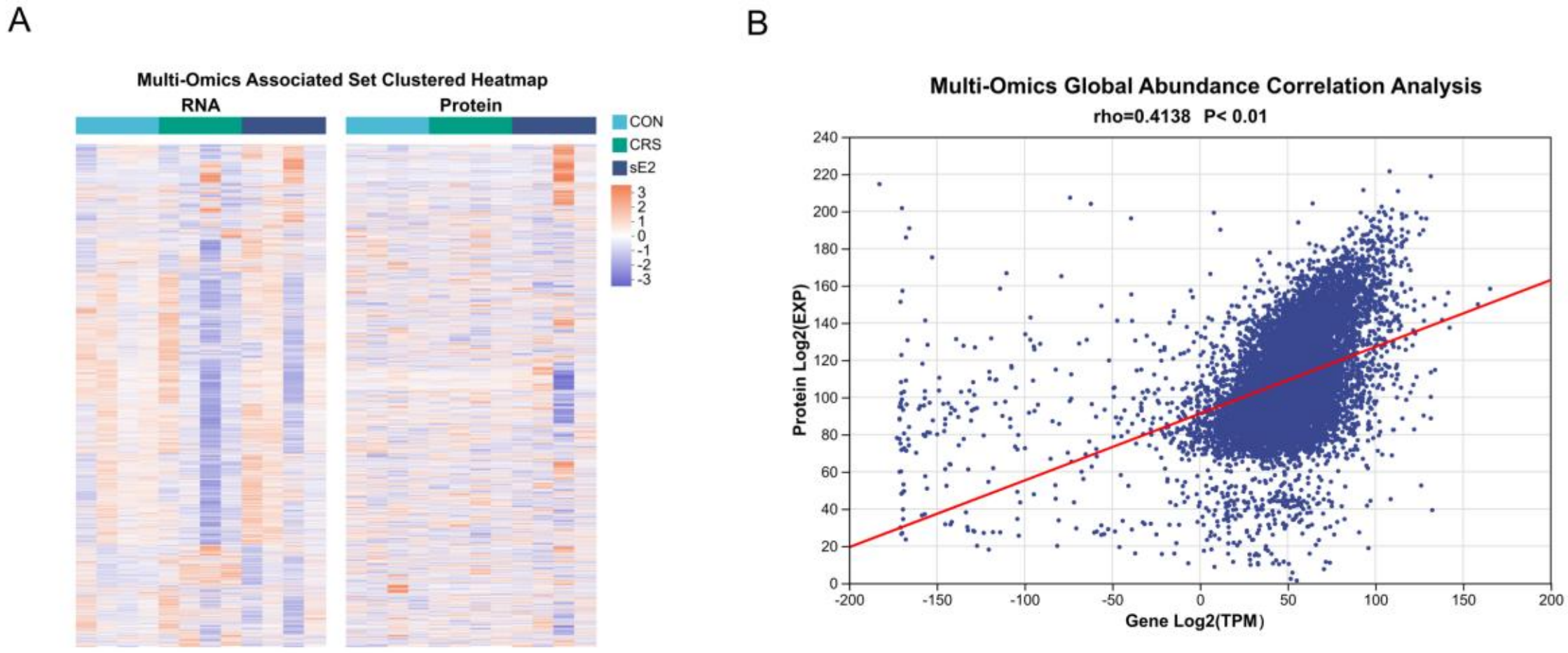


**(A)** Clustered heatmap displaying the relative expression levels (Z-score normalized) of RNA (left) and proteins (right) across Female CON, Female CRS, and Female sE2 groups. **(B)** Global abundance correlation analysis between the transcriptome and proteome (Spearman's rho = 0.4138, $p < 0.01$).

**Supplementary Figure S5. Evaluation of neuroinflammatory factors**

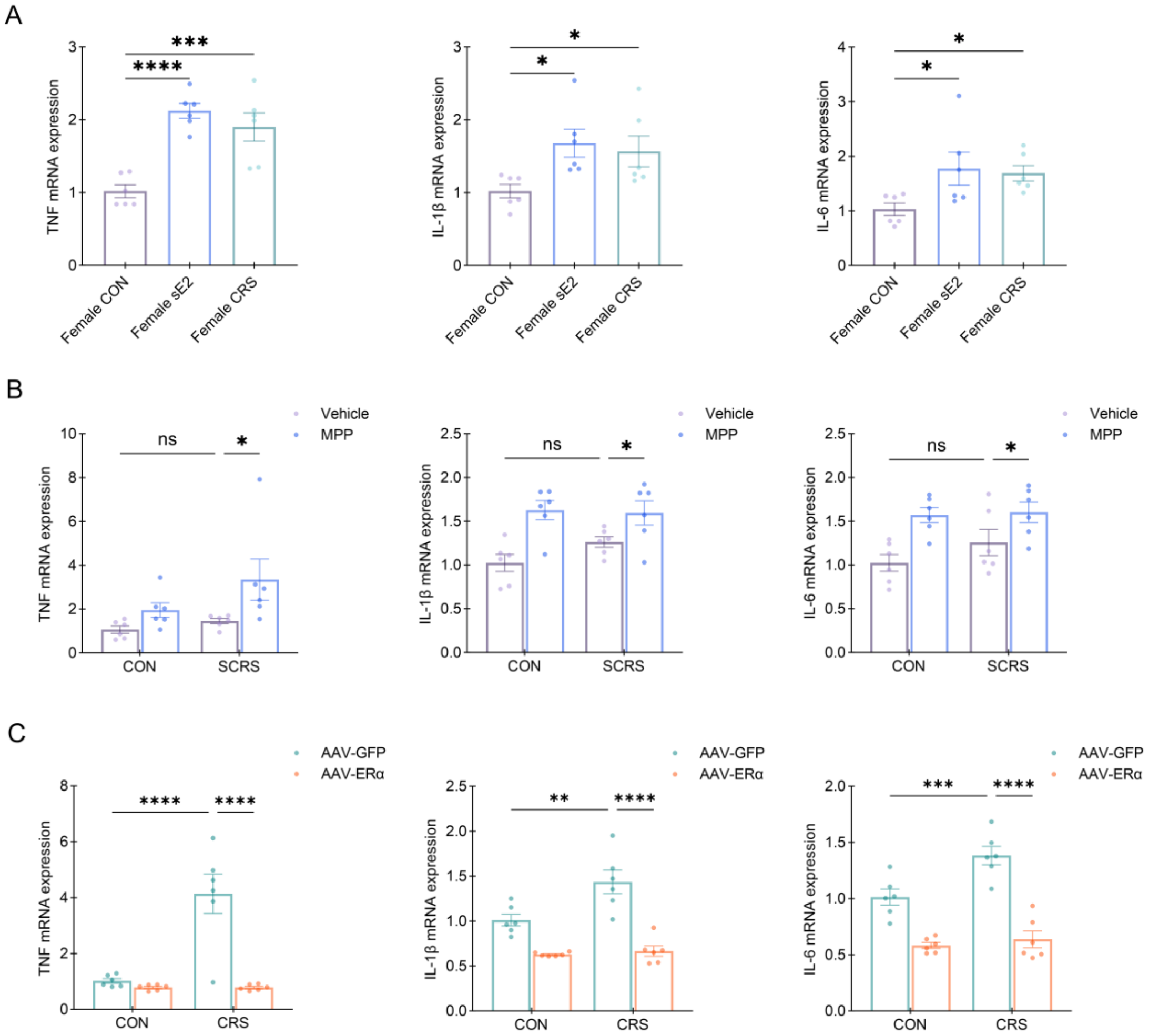


Relative mRNA expression levels of pro-inflammatory cytokines (TNF, IL-1β, IL-6) in the ventral hippocampus of female mouse models. Data are presented as mean ± standard error of mean, *$p < 0.05$, **$p < 0.01$, ***$p < 0.001$, ****$p < 0.0001$.

**Supplementary Data S1. Human MDD GWAS-identified candidate genes**

Data were adapted from Als TD, et al. "Depression pathophysiology, risk prediction of recurrence and comorbid psychiatric disorders using genome-wide analyses," Nat Med. 2023;29(7):1832-1844.

**Supplementary Data S2. Catalog of genes associated with mental disorders from the PsyGeNET database**

Data were adapted from Ma G, et al., "Single-cell transcriptomics reveals Sox6 positive interneurons enriched in the prefrontal cortex of female mice vulnerable to chronic social stress," Mol Psychiatry. 2025;30(11):5058-5071.

# Supplementary Methods

## Supplementary method S1. Preliminary cross-sectional study

### Study Population and Cohort Selection

Our preliminary cross-sectional analysis utilized data from the National Health and Nutrition Examination Survey (NHANES) across three cycles: 2013 – 2016 and 2021 – 2023 [1]. To ensure analytical rigor, participants were selected according to pre-specified inclusion and exclusion criteria. As illustrated in the flowchart (Figure 1a), the initial pool comprised 32,079 participants. After excluding individuals aged < 18 years (n = 11,821), those using exogenous hormones (n = 1,603) or antidepressants (n = 1,003), pregnant or lactating women (n = 263), and participants with incomplete patient health questionnaire-9 items (PHQ-9) or sex hormones data (n = 4,940), 12,449 eligible participants remained [2-4]. To minimize physiological hormonal heterogeneity (menstrual cycle fluctuations and reproductive status), 5,968 female participants were further excluded, resulting in a final core analytical cohort of 6,481 male participants, focusing on the association between serum E2 and depression risk [5-6].

The original NHANES study was approved by the National Center for Health Statistics (NCHS) Research Ethics Review Board, and all participants provided written informed consent. This secondary analysis of de-identified public data was conducted in accordance with the Declaration of Helsinki.

### Measurement of Sex Hormones

Serum concentrations of E2 and testosterone were quantified simultaneously using isotope dilution-liquid chromatography-tandem mass spectrometry (ID-LC–MS/MS). As the international gold standard for hormone measurement, ID-LC–MS/MS provides superior sensitivity and specificity compared to traditional immunoassays, effectively eliminating matrix interference and ensuring high precision. The lower limits of detection (LLOD) for serum E2 and testosterone were 2.994 pg/mL and 0.750 ng/mL, respectively. For values below the LLOD, concentrations were substituted with LLOD divided by the square root of 2 (LLOD/√2) to maintain data integrity for statistical analysis [7-8].

### Assessment of Depressive Symptoms

Depressive symptoms were quantified using the PHQ-9, a validated 9-item self-report screening tool based on the DSM diagnostic criteria. Each item was scored from 0 ("not at all") to 3 ("nearly every day") based on symptoms experienced over the past two weeks, with a total score range of 0–27. According to widely accepted clinical standards, a total score ≥ 10 was utilized as the threshold for

clinically significant depression. To handle missing PHQ-9 data, we followed the NHANES standardized protocol [29]: if at least seven of the nine items were answered (missing ≤ 2 items), the total score was calculated by imputing the missing values with the mean score of the responded items. If more than two items were missing, the participant was excluded from the depression-related analysis to ensure the reliability of the clinical assessment [9-10].

**Definition of Covariates**

A comprehensive set of covariates was extracted and adjusted to control for potential confounding factors: (1) Demographics: Age, race, education, and marital status; (2) Socioeconomic status: family income to poverty (PIR); (3) Lifestyle factors: smoking, drinking, physical activity, and body mass index (BMI); (4) Clinical variables: Medical history of hypertension and diabetes, and self-reported health [11-12].

**Statistical Analysis**

Statistical analyses were performed using R software (version 4.4.1), with $p < 0.05$ considered statistically significant.

Missing values were imputed via multiple imputation by chained equations (mice) using a random forest algorithm [13]. Baseline characteristics across serum E2 quartiles were compared using one-way ANOVA, Kruskal-Wallis, or Chi-square tests as appropriate. The dose-response relationship between log-transformed serum E2 and depression was modeled using restricted cubic splines (RCS) with three knots (10th, 50th, and 90th percentiles); non-linearity was tested via the likelihood ratio test. Multivariable logistic regression estimated odds ratios (ORs) and 95% confidence intervals (CIs) across three models: Model 1: unadjusted; Model 2: adjusted for age, race, BMI, PIR, marital status, education, smoking, drinking, physical activity, self-reported health, hypertension, and diabetes; Model 3: additionally adjusted for log-transformed testosterone to account for androgen-independent effects. Subgroup analyses were performed to assess the consistency of the observed associations across different population characteristics, including age (18–65 vs. ≥ 65 years), BMI (< 30 vs. ≥ 30 kg/m$^2$), race (Non-Hispanic White vs. Other), marital status (Married/Cohabiting vs. Alone), education (High school or less vs. Above high school), PIR (≥ 3.0 vs. < 3.0), physical activity (Moderate-to-vigorous vs. Inactive), and chronic disease status (Hypertension/Diabetes: No vs. Yes). Sensitivity analyses using the non-imputed dataset were conducted to evaluate the robustness of the findings [14-16].

## References


1. Wang Y, Aimuzi R, Nian M, Zhang Y, Luo K, Zhang J. Perfluoroalkyl substances and sex hormones in postmenopausal women: NHANES 2013-2016. Environ Int. 2021;149:106408.
2. Li K, Lyu H, Zhang L, et al. Association between dietary patterns and suicide ideation among depressed adults: Insights from NHANES 2007-2020. J Affect Disord. 2025;377:235-244.
3. Schiller CE, Meltzer-Brody S, Rubinow DR. The role of reproductive hormones in postpartum depression. CNS Spectr. 2015;20(1):48-59.
4. Kroenke K, Spitzer RL, Williams JB. The PHQ-9: validity of a brief depression severity measure. J Gen Intern Med. 2001;16(9):606-613.
5. Randolph JF Jr, Zheng H, Sowers MR, et al. Change in follicle-stimulating hormone and estradiol across the menopausal transition: effect of age at the final menstrual period. J Clin Endocrinol Metab. 2011;96(3):746-754.
6. Yeap BB, Marriott RJ, Dwivedi G, et al. Associations of Testosterone and Related Hormones With All-Cause and Cardiovascular Mortality and Incident Cardiovascular Disease in Men : Individual Participant Data Meta-analyses. Ann Intern Med. 2024;177(6):768-781.
7. Stanczyk FZ, Clarke NJ. Advantages and challenges of mass spectrometry assays for steroid hormones. J Steroid Biochem Mol Biol. 2010;121(3-5):491-495.
8. Huang J, Shi P, Zhao Y, Zhang H, Gao T, Wang X. Associations between smoking, sex steroid hormones, trouble sleeping, and depression among U.S. adults: a cross-sectional study from NHANES (2013-2016). BMC Public Health. 2024;24(1):1541.
9. Accuracy of Patient Health Questionnaire-9 (PHQ-9) for screening to detect major depression: individual participant data meta-analysis. BMJ. 2019;365:l1781.
10. National Center for Health Statistics. 2017-2018 data documentation, codebook, and frequencies: Depression screener (DPQ_J). Hyattsville, MD: U.S. Department of Health and Human Services, Centers for Disease Control and Prevention, 2022.
11. Galvan T, Camuso J, Sullivan K, et al. Association of estradiol with sleep apnea in depressed perimenopausal and postmenopausal women: a preliminary study. Menopause. 2017;24(1):112-117.
12. Li D, Sun T, Tong Y, et al. Gut-microbiome-expressed 3β-hydroxysteroid dehydrogenase

degrades estradiol and is linked to depression in premenopausal females. Cell Metab. 2023;35(4):685-694.e5.

13. Patrician PA. Multiple imputation for missing data. Res Nurs Health. 2002;25(1):76-84.

14. Shen W, Zhang W, Ye M, et al. Profile of anxiety symptoms in adolescents in Zhejiang, China. J Affect Disord. 2026;395(Pt B):120787.

15. Wang R, Lagakos SW, Ware JH, Hunter DJ, Drazen JM. Statistics in medicine--reporting of subgroup analyses in clinical trials. N Engl J Med. 2007;357(21):2189-2194.

16. White IR, Royston P, Wood AM. Multiple imputation using chained equations: Issues and guidance for practice. Stat Med. 2011;30(4):377-399.

**Supplementary method S2. Sample Size and Exclusion Criteria**

All sample sizes were determined by power analysis. All experiments were independently repeated at least 3 times. A random subset of behaviorally validated mice was used for molecular analyses. Female mice in estrus were excluded except for the sE2-treated group. Mice that died, showed abnormal behavior, or had incorrect viral injections were also excluded. Final effective sample sizes:

1. Male CON (11), Male CRS (11), Female CON (12), Female CRS (11);
2. Female CON (12), Female sE2 (20), Female CRS (18);
3. CON+Vehicle (9), CON+MPP (8), SCRS+Vehicle (9), SCRS+MPP (10);
4. CON+GFP (16), CON+ERα (18), CRS+GFP (15), CRS+ERα (16).

**Supplementary method S3. Integrative Multi-Omics Pipeline and Translational Bioinformatics Analyses**

**Rank-Rank Hypergeometric Overlap**

Transcriptomic data and corresponding phenotypic information (accession number GSE102556) were retrieved from the NCBI Gene Expression Omnibus (GEO) database using the GEOquery package in the R programming environment [1]. Based on brain region annotations, samples derived from the ventral subiculum were selected and subsequently stratified by sex. Within each cohort, the log2 fold change and p-value for each gene were calculated using a standardized differential expression analysis pipeline [2]. Subsequently, the Rank-Rank Hypergeometric Overlap (RRHO) method was employed to compare the differential expression profiles across the human sex cohorts without relying on predefined significance thresholds, followed by a cross-species comparative analysis against mouse ventral hippocampal transcriptomic data [3]. First, genes were ranked based on the metric −log10(p-value) × sign(log2 fold change), ensuring that the most significantly upregulated and downregulated genes were positioned at opposite ends of the ranked list. Next, the analysis was conducted using the RRHO package with a sliding window step size set to 200. Hypergeometric tests were applied to evaluate the statistical significance of gene overlap between the two ranked lists at various cutoffs. Finally, RRHO heatmaps were generated to visualize the comparative results, where regions of significant enrichment indicate either concordant or discordant regulatory trends between the gene sets [4].

From the RRHO2 output, we extracted the gene sets corresponding to the maximally significant overlap (MSO) within the downregulated-downregulated (DD) quadrants for each pairwise comparison (sE2 vs. Human MDD and sE2 vs. CRS). To identify a highly conserved transcriptional signature, a consensus core was defined by intersecting these two pairwise DD gene lists. Functional enrichment analysis was subsequently performed on the consensus genes using the R package clusterProfiler. Gene Ontology (GO) terms were further identified, with *p*-values adjusted using the Benjamini-Hochberg (BH) method.

**Gene Set Enrichment Analysis**

To further elucidate the biological functions and signaling pathways associated with depression, Gene Set Enrichment Analysis (GSEA) was performed using the clusterProfiler R package [5]. Instead of focusing solely on individual differentially expressed genes, GSEA was employed to evaluate whether

predefined sets of genes exhibited statistically significant, cumulative differences between groups. We conducted a comprehensive functional enrichment analysis across four primary libraries: the MSigDB Hallmark gene sets, Gene Ontology (GO), Kyoto Encyclopedia of Genes and Genomes (KEGG), and the Reactome Pathway Database. All detected genes were pre-ranked based on the ranking metric −log10(p-value) × sign(log2 Fold Change) derived from the differential expression analysis. The Enrichment Score (ES) was calculated using a weighted Kolmogorov-Smirnov statistic, followed by 1,000 permutations to determine statistical significance. Pathways with a nominal $p$-value < 0.05, and a False Discovery Rate (FDR) q-value < 0.25 were considered significantly enriched [6].

**Identification of Differentially Expressed Genes and Proteins**

To identify molecular signatures associated with depression and the effects of estrogen signaling, differential expression analysis was performed. For transcriptomic analysis, two sets of criteria were applied: (1) In the initial experimental set (Female CON, Female CRS, and Female sE2), differentially expressed genes (DEGs) were identified using a threshold of absolute fold change (FC) > 1.3 with a raw $p$-value < 0.05, using the Female CON group as the reference. (2) In the intervention set (CRS+ERα vs. CRS+GFP), a more stringent statistical threshold was employed, where DEGs were identified using a threshold of absolute FC > 1.3 and an adjusted $p$-value ($p$-adj) < 0.05, with the CRS+GFP group serving as the control. For proteomic analysis, differentially expressed proteins (DEPs) were screened between the Female CON, CRS, and sE2 groups. Proteins with an FC > 1.2 or < 0.83 and a raw $p$-value < 0.05 were considered significantly altered, relative to the Female CON group. All fold changes and statistical significances were calculated via the DESeq2 and limma R packages for transcriptomics and proteomics, respectively [7-8].

**Transcription Factor Enrichment and Upstream Regulator Identification**

To investigate the potential upstream transcriptional regulators driving the observed molecular dysregulation, transcription factor (TF) enrichment analysis was performed using the ChEA3 (ChIP-Enrichment Analysis 3) platform. The 147 convergent DEGs and 94 convergent DEPs, representing the shared signatures between the CRS and sE2 models, were utilized as input gene sets. We specifically queried the Literature ChIP-seq library within ChEA3 to identify TFs with significantly overrepresented binding sites among these convergent targets. TFs were ranked based on their statistical significance, calculated as the -log10 (Fisher's Exact Test $p$-value). This integrative analysis aimed to identify core

regulatory nodes, such as ESR1, that may coordinate the transcriptomic and proteomic alterations in the vHPC across different experimental conditions [9].

**Integrated Multi-Omics Analysis**

To identify robust biological signatures shared between the transcriptome and proteome, an integrated meta-analysis was conducted. First, a global abundance correlation was performed by calculating the Spearman correlation coefficient (rho) between gene expression levels (Log2 TPM) and protein abundance levels (Log2 EXP). For the integrative functional analysis, we employed Stouffer's Z-score method to combine the results from the transcriptomic and proteomic datasets. Specifically, the p-values and the direction of enrichment (Normalized Enrichment Scores, NES) from the individual GSEA of each omics level were converted into standard Z-scores. These Z-scores were then integrated using a weighted approach to calculate a Meta-Z-score for each biological pathway. This meta-analysis allowed us to identify convergent multi-omics pathways that were consistently dysregulated at both the mRNA and protein levels across the CRS and sE2 models [10-11]. The resulting integrated scores were visualized using bar plots, and the expression patterns of the top-ranked convergent multi-omic genes were further validated and visualized using dot plots. Additionally, all abundance data for the multi-omics associated sets were Z-score normalized for clustered heatmap visualization.

**Functional Enrichment Analysis (GO and KEGG)**

To further characterize the functional impact of ERα (ESR1) overexpression in the vHPC, GO and KEGG enrichment analyses were performed on the DEGs identified from the CRS+ERα vs. CRS+GFP comparison. The GO analysis categorized the DEGs into three primary functional domains: Biological Process (BP), Cellular Component (CC), and Molecular Function (MF). The KEGG pathway analysis was employed to identify the biological signaling pathways and metabolic processes significantly enriched with these ESR1-regulated genes. Statistical significance for the enrichment was determined based on a raw $p$-value < 0.05 or a Benjamini-Hochberg adjusted $p$-value ($p$-adj) < 0.05 [12-13].

**Clinical and Translational Relevance Analysis**

To evaluate the clinical and translational significance of the ERα (ESR1)-regulated molecular signatures in the vHPC, three layers of integrative analyses were performed.

Firstly, after filtering for protein-coding transcripts and performing mouse-to-human ortholog mapping, a cross-model validation was conducted using a Venn diagram to identify the overlap between ESR1-regulated DEGs and the convergent DEGs previously identified from the CRS and sE2 models

[14]. Secondly, to assess the genetic relevance, these prioritized orthologs were overlapped with Genome-Wide Association Study (GWAS) hits linked to depression [15]. Thirdly, the refined ESR1-regulated gene set was queried against the PsyGeNET database to explore its association with a broad spectrum of psychiatric disorders [16].

Finally, to translate these molecular mechanisms into potential pharmacological interventions, a Connectivity Map (CMap) analysis was employed. The ERα-regulated gene signature was utilized to query the database to identify top small-molecule compounds (candidate therapy mimetics) capable of inducing transcriptomic profiles highly similar to the ERα-rescue effect [17].

**References**

1. Davis S, Meltzer PS. GEOquery: a bridge between the Gene Expression Omnibus (GEO) and BioConductor. Bioinformatics. 2007;23(14):1846-1847.
2. Labonté B, Engmann O, Purushothaman I, et al. Sex-specific transcriptional signatures in human depression. Nat Med. 2017;23(9):1102-1111.
3. Plaisier SB, Taschereau R, Wong JA, Graeber TG. Rank-rank hypergeometric overlap: identification of statistically significant overlap between gene-expression signatures. Nucleic Acids Res. 2010;38(17):e169.
4. Piron A, Szymczak F, Papadopoulou T, et al. RedRibbon: A new rank-rank hypergeometric overlap for gene and transcript expression signatures. Life Sci Alliance. 2023;7(2):e202302203.
5. Wu T, Hu E, Xu S, et al. clusterProfiler 4.0: A universal enrichment tool for interpreting omics data. Innovation (Camb). 2021;2(3):100141.
6. Subramanian A, Tamayo P, Mootha VK, et al. Gene set enrichment analysis: a knowledge-based approach for interpreting genome-wide expression profiles. Proc Natl Acad Sci U S A. 2005;102(43):15545-15550.
7. Love MI, Huber W, Anders S. Moderated estimation of fold change and dispersion for RNA-seq data with DESeq2. Genome Biol. 2014;15(12):550.
8. Ritchie ME, Phipson B, Wu D, et al. limma powers differential expression analyses for RNA-sequencing and microarray studies. Nucleic Acids Res. 2015;43(7):e47.
9. Keenan AB, Torre D, Lachmann A, et al. ChEA3: transcription factor enrichment analysis by

orthogonal omics integration. Nucleic Acids Res. 2019;47(W1):W212-W224.

10. Whitlock MC. Combining probability from independent tests: the weighted Z-method is superior to Fisher's approach. J Evol Biol. 2005;18(5):1368-1373.
11. Hwang D, Rust AG, Ramsey S, et al. A data integration methodology for systems biology. Proc Natl Acad Sci U S A. 2005;102(48):17296-17301.
12. Gene Ontology Consortium. The Gene Ontology resource: enriching a GOld mine. Nucleic Acids Res. 2021;49(D1):D325-D334.
13. Kanehisa M, Furumichi M, Sato Y, Kawashima M, Ishiguro-Watanabe M. KEGG for taxonomy-based analysis of pathways and genomes. Nucleic Acids Res. 2023;51(D1):D587-D592.
14. Chen H, Boutros PC. VennDiagram: a package for the generation of highly-customizable Venn and Euler diagrams in R. BMC Bioinformatics. 2011;12:35.
15. Als TD, Kurki MI, Grove J, et al. Depression pathophysiology, risk prediction of recurrence and comorbid psychiatric disorders using genome-wide analyses. Nat Med. 2023;29(7):1832-1844.
16. Gutiérrez-Sacristán A, Grosdidier S, Valverde O, et al. PsyGeNET: a knowledge platform on psychiatric disorders and their genes. Bioinformatics. 2015;31(18):3075-3077.
17. Subramanian A, Narayan R, Corsello SM, et al. A Next Generation Connectivity Map: L1000 Platform and the First 1,000,000 Profiles. Cell. 2017;171(6):1437-1452.e17.
